# Potential causes and consequences of rapid mitochondrial genome evolution in thermoacidophilic *Galdieria* (Rhodophyta)

**DOI:** 10.1186/s12862-020-01677-6

**Published:** 2020-09-07

**Authors:** Chung Hyun Cho, Seung In Park, Claudia Ciniglia, Eun Chan Yang, Louis Graf, Debashish Bhattacharya, Hwan Su Yoon

**Affiliations:** 1 Department of Biological Sciences, Sungkyunkwan University, Suwon, 16419 South Korea; 2 Department of Environmental, Biological and Pharmaceutical Science and Technologies, University of Campania Luigi Vanvitelli, 81100 Caserta, Italy; 3 Marine Ecosystem Research Center, Korea Institute of Ocean Science and Technology, Busan, 49111 South Korea; 4 Department of Biochemistry and Microbiology, Rutgers University, New Brunswick, 08901 USA

**Keywords:** Cyanidiophyceae, Extremophile, Mitogenome evolution, Protein divergence, Mitochondrial DNA replication

## Abstract

**Background:**

The Cyanidiophyceae is an early-diverged red algal class that thrives in extreme conditions around acidic hot springs. Although this lineage has been highlighted as a model for understanding the biology of extremophilic eukaryotes, little is known about the molecular evolution of their mitochondrial genomes (mitogenomes).

**Results:**

To fill this knowledge gap, we sequenced five mitogenomes from representative clades of Cyanidiophyceae and identified two major groups, here referred to as *Galdieria*-type (*G*-type) and *Cyanidium*-type (*C*-type). *G*-type mitogenomes exhibit the following three features: (i) reduction in genome size and gene inventory, (ii) evolution of unique protein properties including charge, hydropathy, stability, amino acid composition, and protein size, and (iii) distinctive GC-content and skewness of nucleotides. Based on GC-skew-associated characteristics, we postulate that unidirectional DNA replication may have resulted in the rapid evolution of *G*-type mitogenomes.

**Conclusions:**

The high divergence of *G*-type mitogenomes was likely driven by natural selection in the multiple extreme environments that *Galdieri*a species inhabit combined with their highly flexible heterotrophic metabolism. We speculate that the interplay between mitogenome divergence and adaptation may help explain the dominance of *Galdieri*a species in diverse extreme habitats.

## Background

Acidic hot springs environments can place severe stresses on unicellular organisms due to the high temperature, low pH, and elevated heavy-metal concentrations [1]. Most hot springs biodiversity is comprised of prokaryotes, with only a few eukaryotes able to compete successfully in these extreme habitats [2, 3]. The unicellular red algal class, Cyanidiophyceae, is a well-known group of extremophilic eukaryotes that thrives in acidic (pH 0.5–3.0) and high temperature (50–55 °C) habitats [4, 5]. Cyanidiophyceae have traditionally been reported from volcanic regions around the world [6–8], whereas some mesophilic species (e.g., *Cyanidium chilense*) are found in moderately acidic caves (pH 5–7) around volcanic regions in Chile, Italy, France, Israel, and Turkey [4, 9–12]. Cyanidiophyceae is the earliest diverging red algae lineage, having split from other Rhodophyta about 1.5 billion years ago [5, 13]. Due to limited ultrastructural differences and the “simple” morphology of cyanidiophycean cells, it is difficult to discriminate between species using microscopy. This has led to the hypothesis of cryptic diversity based on molecular phylogenetic studies [4, 14, 15]. Moreover, incongruent topologies or unresolved relationships of Cyanidiophyceae are reported in most molecular phylogenetic studies using single (e.g., *rbc*L, SSU rRNA) or a few genes (e.g., *rbc*L + *psa*A + *psb*A) [4, 10, 14, 16–18].

Most Cyanidiophyceae are photoautotrophs and inhabit niches exposed to sulfur fumes, however the genus *Galdieria* also occupies endolithic and interlithic habitats [4, 16], and is therefore more exposed to microenvironmental fluctuations [e.g., diverse microhabitats, desiccation, a broad range of acidity (pH 0.5–6.0), temperature (18–55 °C), and salinity (4–10%)] [4, 8, 10, 19–25] . Members of this genus are also grow mixotrophically, able to utilize > 50 carbon sources [26]. From a genomic point of view, previous studies have demonstrated that *Galdieria* species contain a variety of horizontally transferred genes that confer adaptation for specific habitats (e.g., endolithic) [27–29]. Although Cyanidiophyceae genomes are significantly reduced in size (< 20 Mbp), *Galdieria* species retain a nearly intact spliceosomal machinery and have a relatively large number of introns that likely play a role in regulating the stress response [25].

*Galdieria* species have greatly reduced mitochondrial genomes and these genes have the highest substitution rate among all red algae likely due to the polyextremophilic lifestyle [30]. However, only two published (*Cyanidioschyzon merolae* 10D and *Galdieria sulphuraria* 074 W) and one unpublished (Cyanidiophyceae sp. MX-AZ01) mitochondrial genomes are available to date [30, 31]. To better understand cyanidiophycean mitochondrial genome evolution, we generated five new complete mitogenomes that represent all of the major clades, including the three genera of Cyanidiophyceae. Among them, three *Galdieria* mitogenomes are not only highly reduced in size, but also substantially differ in protein characteristics compared to Cyanidiophyceae and other red algae. Based on GC-skewness and other mitogenomic features, we postulate that a unique replication system may exist in *Galdieria* species.

## Results and discussion

To confirm the phylogenetic position of the eight strains under study, we used the *rbc*L phylogeny to identify the major groups of Cyanidiophyceae (Fig. S1). The taxon-rich (269 taxa) *rbc*L tree showed five major cyanidiophycean groups: 1) *Cyanidium chilense* assemblage (*Cd. chilense*; known as mesophilic *Cyanidium* sp.) [12], 2) *Cyanidium caldarium* (*Cd. caldarium*), 3) *Galdieria sulphuraria* assemblage (*G. sulphuraria*), 4) *Cyanidioschyzon merolae* (*Cz. merolae*), and 5) *Cyanidiococcus yangmingshanensis* assemblage (*Cc. yangmingshanensis*; known as *Galdieria maxima*) [32], which is consistent with previous studies [14, 18]. The eight strains used in this study adequately represent the diversity of the five major groups of Cyanidiophyceae (see in Fig. S1).

The general characteristics of the eight mitogenomes were compared including five new and three published datasets (Table 1) [30, 31]. These mitogenomes are clearly divided into two types as *Cyanidium*-type and *Galdieria*-type based on mitogenome features (e.g., genome size, genome structure, number of genes, skewness of nucleotides). *Cyanidium*-type (*C*-type) is comprised of five taxa (mesophilic *Cd. chilense* Sybil Cave*, Cd. caldarium, Cz. merolae*, *Cc. yangmingshanensis,* and Cyanidiophyceae sp. MX-AZ01), and the *Galdieria*-type (*G*-type) is comprised of three taxa (*G. phlegrea*, *G. sulphuraria* 108.79 E11 and 074 W).

**Table 1:** General characteristics of Cyanidiophyceae mitogenomes

Type	***Cyanidium***-type (***C***-type)					***Galdieria***-type (***G***-type)		
Species*	CZME 10D	CYSP MX-AZ01	CCYA 8.1.23 F7	CDCA ACUF 019	CDCH Sybil Cave	GAPH DBV 009	GASU 074 W	GASU 108.79 E11
**Genome Size (bp)**	32,211	32,620	32,386	34,207	33,039	21,792	21,428	21,611
**GC-content (%)**	27.1	26.7	26.4	25.9	44.5	41.4	44.0	41.8
**GC-skew**	0.06	0.03	0.03	0.02	0.01	0.71	0.74	0.66
**AT-skew**	0.01	0.02	0.03	0.03	0.03	0.25	0.25	0.29
**Number of Genes**	64	61	62	61	61	26	27	27
**Non-coding Region (%)**	5.20	6.50	5.13	10.64	6.37	17.55	15.55	16.49
**NCBI Accession Number**	NC_000887	KJ569774	MT270119 (this study)	MT270118 (this study)	MT270117 (this study)	MT270116 (this study)	NC_024666	MT270115 (this study)

*abbreviations: *CZME* (*Cyanidioschyzon merolae*), *CYSP* (Cyanidiophyceae sp.), *CCYA* (*Cyanidiococcus yangmingshanensis*), *CDCA* (*Cyanidium caldarium*), *CDCH* (*Cyanidium chilense*), *GAPH* (*Galdieria phlegrea*), *GASU* (*Galdieria sulphuraria*)

### General features of Cyanidiophyceae mitogenomes

The mitogenome size of *Cyanidium*-type (*C*-type) is roughly 10 kbp larger than in *Galdieria*-type (*G*-type) (see Table 1). This size difference between the two types is explained by a larger number of tRNA encoding genes and CDSs in the *C*-type (33–35 CDS, 23–25 tRNA genes) than in the *G*-type (18 CDS, 6–7 tRNA genes). SSU (rrs) and LSU (rrl) rRNAs are present in all species, yet 5S rRNA sequences were identified only in the *C*-type mitogenomes, except for *Cyanidium caldarium*. This finding is congruent with former results that showed the 5S rRNA gene in red algal mitogenomes to be pseudogenized or lost outright [33]. Portions of non-coding regions in *C*-type (5.13–10.64%) are smaller than those of *G*-type (15.90–17.64%), mostly due to the existence of unknown sequence insertions (2–3 kbp length, *ymf*39-*cox*1 region).

**Fig. 1:**
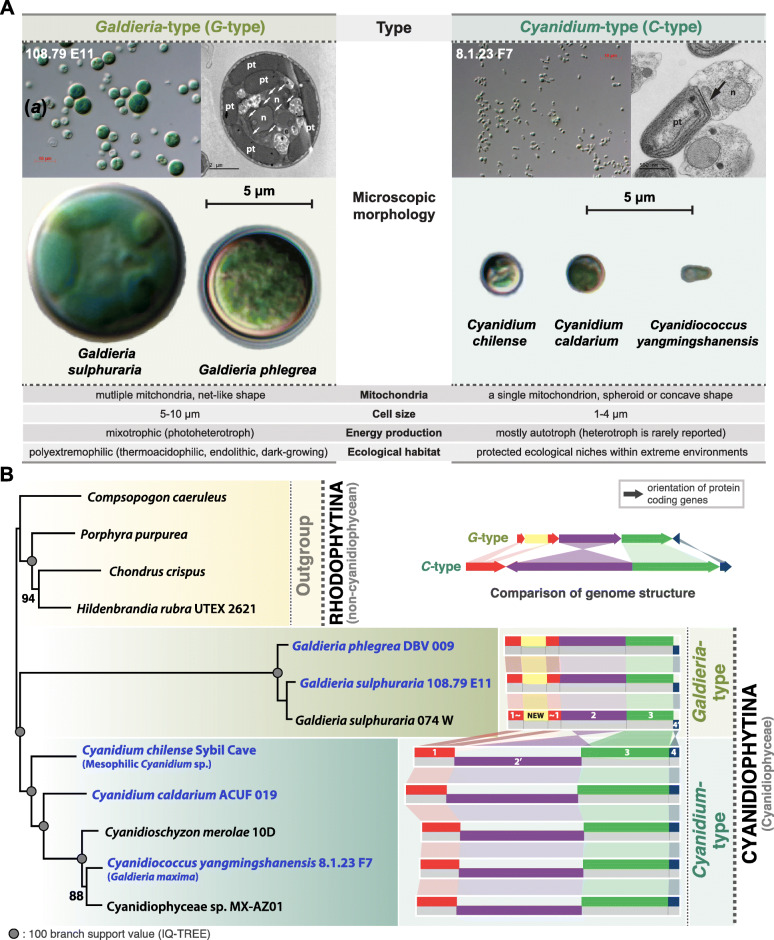
**Overview of the major characteristics of Cyanidiophyceae and its phylogeny**. **a** Comparison of key characteristics of the *Cyanidium*-type and *Galdieria*-type species showing two different types of cyanidiophycean cells. Based on existing studies, key characteristics were summarized in this figure with n: nucleus, pt: plastid, and arrow: mitochondria. **b** Maximum-likelihood phylogeny using a concatenated 32-protein alignment of 12 mitochondrial genomes. Four non-cyanidiophycean species were chosen as the outgroup. The simplified genome structure of cyanidiophycean mitochondria is illustrated next to the phylogenetic tree

**Fig. 2:**
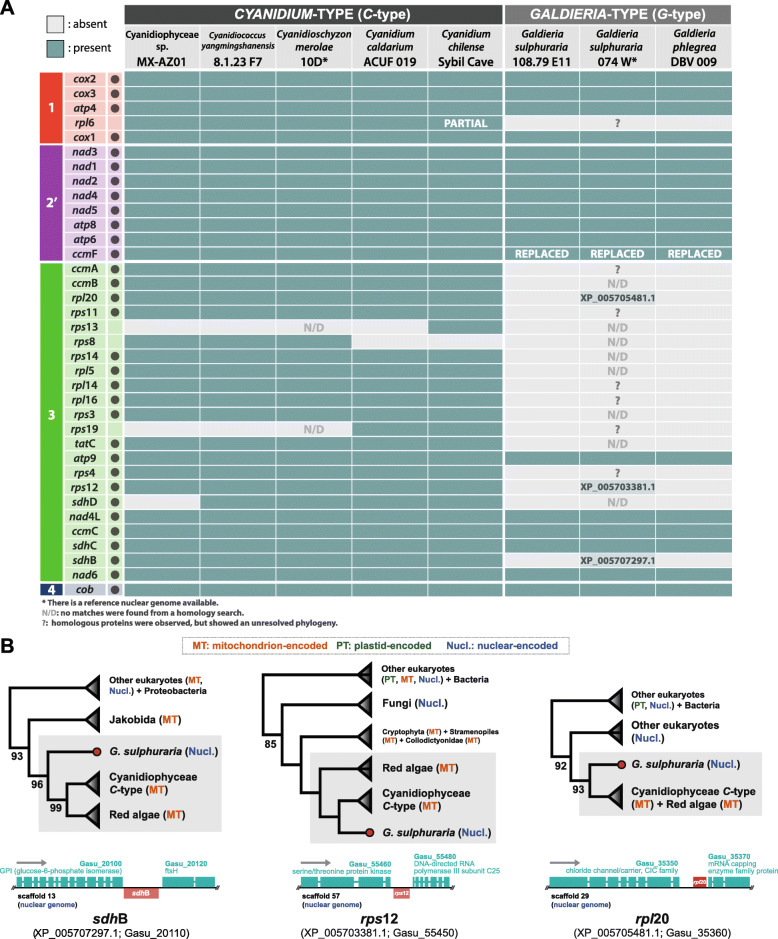
**Mitochondrial gene content in cyanidiophycean mitogenomes and the phylogeny of three EGT-derived genes. a** The presence and absence of 36 mitochondrial genes in Cyanidiophyceae is shown. Black dots indicate genes used for concatenated dataset phylogenetic analysis. Each number in colored box represents different gene synteny and reversed orientation is indicated by the prime mark (’). **b** The phylogeny of three EGT-derived genes and their location in the *Galdieria sulphuraria* 074 W genome. Bootstrap values > 90% support merged clades (triangles) and bootstrap support values < 50% are now shown

Before comparing the genome structures, we aligned all genomes starting from the start codon of the *cox*2 gene. For comparative genome structure analysis, Mauve Genome Alignment v2.2.0 [34] with default settings and UniMoG using the DCJ model [35] were used. Comparison of genome structure reveals that Cyanidiophyceae mitogenomes are divided into the four synteny blocks shown in Figs. 1, 2; *cox*2-*cox*1 region (red block), *nad*3-*yej*R region (purple block), rDNA region including adjacent genes (green block), and *cob* region (blue block). These synteny blocks are conserved between the *Cyanidium*-type and *Galdieria*-type, however, there is a unique 2–3 kbp sequence insertion (yellow block) found between *atp*4 and *cox*1 genes (red block) only in the *Galdieria*-type. An open reading frame (i.e., *orf*181 gene in *G. sulphuraria* 074 W) is present in this inserted region, but none of these sequences have significant hits in the NCBI or UniProt databases. These four, shared synteny blocks are identical in each type, however, two inversions and one deletion are found between the types; 1) the *Cyanidium*-type has a reversed *nad*3-*yej*R coding region (purple block) order, which is in the forward direction in *Galdieria*-type. 2) *cob* gene (blue block) is a reverse-complemented relative to *Cyanidium*-type (Figs. 1, 2). There is significant gene loss in the green synteny block of *Galdieria*-type. The 16–17 kbp of the rDNA region of *Cyanidium*-type includes 18–20 coding genes, two rDNAs, and two tRNAs, whereas the *Galdieria*-type (5–6 kbp length) is composed of five genes, two rDNAs, and 18 tRNAs.

Based on our observations and previous work, *C*-type and *G*-type are recognized not only using mitogenome characteristics, but also on the basis of morphological characteristics, cellular features, and ecological habitats (Fig. 1a) [4, 6–8, 10, 14, 16, 21–23, 36, 37]. The general morphology of *G*-type differs from *C*-type with regard to a larger cell size (*G*-type: 5–10 μm, *C*-type: 1–4 μm), presence of a vacuole, organelle shape (e.g., branched mitochondria, multi-lobed plastid), and a simple spherical morphology, whereas *C*-type species show more diverse cell shapes (e.g., spherical, oval, club-shaped) (Fig. 1a) [4, 8, 10, 19–22, 36, 38]. Particularly for mitochondrial features, *G*-type *Galdieria sulphuraria* contains several mitochondria per cell that have a net-like structure, whereas *C*-type *Cd. caldarium* and *Cz. merolae* contain a single mitochondrion in a cell [38]. In our transmission electron microscopic observation, multiple mitochondria are identified in *G*-type *G. sulphuraria* 108.79 E11, whereas a single spheroid mitochondrion is found in *C*-type *Cc. yangmingshanensis* 8.1.23 F7 (Figs. 1a). The ecophysiological niche of *G*-type species is more diverse than *C*-type species and includes hydrothermal regions, acid mine drainage sites, endolithic environments, and surfaces of burning coal [4, 10, 16–18, 24]. In contrast, the habitats of *C*-type species are more ecologically “protected” such as in ditches and streams around hot springs that exhibit lower temperature fluctuations [4, 10, 16–18, 21, 24]. These habitat differences suggest that *G*-type species have radiated in more diverse environments that *C*-type species.

### *Cyanidium*-type and *Galdieria*-type mitogenomes resolved using phylogenetic analysis

Phylogenetic analysis also supports the recognition of the *C*-type and *G*-type mitogenomes. The concatenated protein ML phylogeny using 36 mitochondrial genes resolves the *C*-type and *G*-type with full bootstrap support (see Fig. 1b). Within the *C*-type, the mesophilic *Cd. chilense* Sybil Cave diverged first, followed by *Cd. caldarium* ACUF 019. *Cz. merolae* 10D are grouped together with the monophyletic clade of the *Cc. yangmingshanensis* 8.1.23 F7 + Cyanidiophyceae sp. MX-AZ01. Our current mitogenome data resolve the internal relationships within the Cyanidiophyceae, in particular, the positions of the mesophilic *Cd. chilense* Sybil Cave and *Cd. caldarium* ACUF 019 that have been poorly resolved until now (Fig. S2). In this mitogenome data analysis, however, we observed an extraordinarily long internal branch of the *G*-type (Fig. 1b), which implies high divergence when compared to *C*-type species or unidentified/extinct genetic diversity in the *G*-type lineages. In addition, we tested individual gene phylogenies to see the consistency compared to concatenated gene tree in Supplementary Information 1. Applying variable mitochondrial gene datasets, we were able to resolve the phylogenetic relationships among major cyanidiophycean clades, in particular the unsettled position of the mesophilic *Cyanidium* clade (Fig. S2).

### Different CDS content between *Cyanidium*-type and *Galdieria*-type mitogenomes: gene loss and transfer

After the recognition of two different groups in Cyanidiophyceae, we focused on mitogenomes gene gains and losses. Comparison of CDS content between the two different types revealed that one-half of mitochondrion-encoded genes in *C*-type mitogenomes are missing in the *G*-type, and changed in synteny regarding the green block (Fig. 2a) [i.e., losses of all ribosomal protein genes (*rps*, *rpl*) and a few core genes (*ccm*A,B and *sdh*B,D)]. To examine endosymbiotic gene transfer (EGT) from mitochondrial to the nuclear genome for these missing genes in *G*-type, we searched 18 homologous genes in the two available nuclear genomes of *Cz. merolae* 10D and *G. sulphuraria* 074 W [28, 39]. Ten genes were identified from the nuclear genome of *G. sulphuraria* 074 W, but we were not able to identify eight mitochondrial genes (‘N/D’ in Fig. 2a) that may indicate outright gene losses in the mitogenome, although it is not possible to rule out issues related to low-quality genome data. Other explanations for missing genes is either high diversification after gene transfer to the nucleus or degeneration of genes from the mitochondria [40]. Out of twelve ribosomal proteins, only *rps*12 and *rpl*20 were found in the *G. sulphuraria* 074 W nuclear genome. These nuclear-encoded *rps*12 and *rpl*20 genes were not grouped together but rather located in two different scaffolds, 57 and 29, respectively (Fig. 2b), whereas most ribosomal protein encoding genes are located in a single syntenic block in *C*-type mitogenomes (*ccm*A-*nad*6; green block in Fig. 2a). We could not detect the remaining ribosomal protein encoding genes, instead, we found six nuclear-encoded homologs of ribosomal protein (*rpl*6, *rpl*14, *rpl*16, *rps*4, *rps*11, *rps*19) in the genome (see question marks in Fig. 2a). With the *ccm*F gene (see Supplementary Information 2 for details), the origins of six homologous genes of ribosomal protein were unclear based on phylogenetic analyses due to low bootstrap support values.

It is unlikely that mitochondrial translation would function properly without a complete set of ribosomal subunit proteins, therefore, the nuclear-encoded homologs could “compensate” for gene losses (e.g., 16–19 tRNAs loss in *G*-type mitogenomes). Meanwhile, the homologs of *rps*13 and *rps*19 were not detected from the *Cz. merolae* 10D but were found in the early branched, mesophilic *Cd. chilense*, suggesting independent gene losses in both *C*-type and *G*-type mitogenomes. It was possible to detect a plastidial-copy, nuclear-copy (host-derived), or other (e.g., unknown sources) ribosomal proteins from homologous searches, which implies the possibility to translocate ribosomal subunits from various origins (e.g., nuclear, plastid, other bacteria) into mitochondria as suggested in previous studies [41–43].

### Changes in amino acid composition in *Galdieria*-type mitochondrial genes

**Fig. 3:**
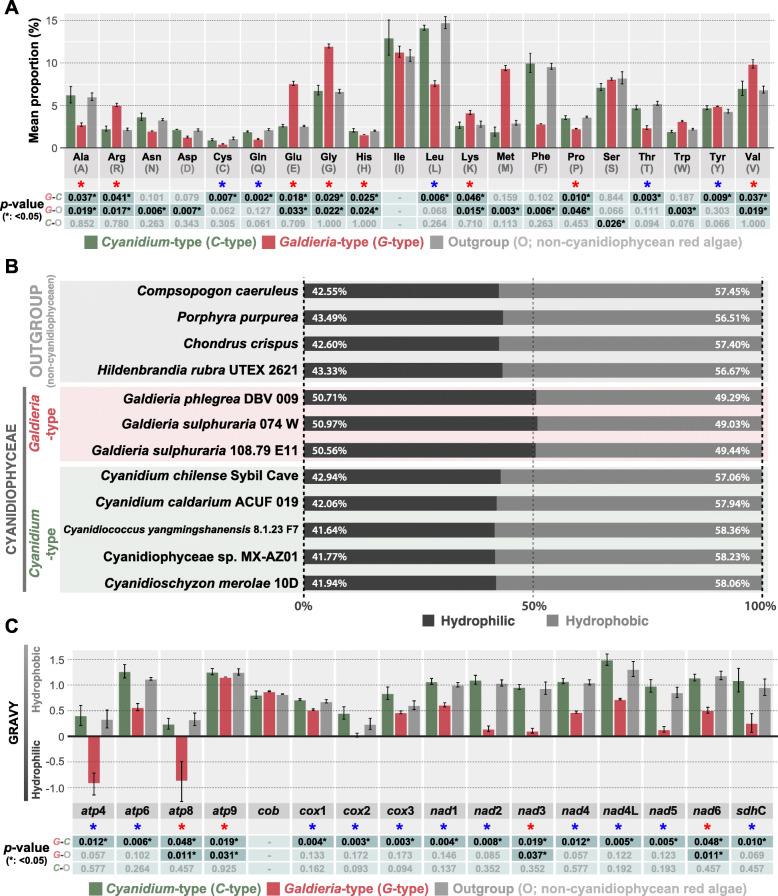
**Amino acid composition of conserved sites and their properties.** A total of 16 mitochondrial genes from 13 species was chosen for this analysis. Blue asterisks indicate a statistically significant difference between *G*-type and *C*-type. The red asterisks indicate a significant difference between *G*-type and *C*-type, and *G*-type and non-cyanidiophycean red algae (see Tables S6, S7). **a** Relative amino acid compositions of conserved mitochondrial genes. **b** Relative hydrophilic and hydrophobic composition of conserved mitochondrial genes. **c** Comparison of hydropathy in 16 conserved mitochondrial proteins

Enzymes from extremophilic organisms have high thermostability, more charged amino acid composition, and reduced hydrophobic surfaces to withstand extreme temperatures and pH [44, 45]. Furthermore, proteins of thermophilic species are shorter in length than those in their mesophilic counterparts [46]. To investigate protein characteristics, we compared protein charge, hydropathy, and stability in 16 genes (genes in Fig. 3) that are retained in all red algal mitogenomes (i.e., *G*-type, *C*-type, non-cyanidiophycean red algae) used in this study.

Amino acid composition of these 16 conserved mitochondrial genes shows that 13 out of 20 amino acids are significantly different (asterisks in Fig. 3a) between *G*-type and *C*-type and this resulted in a difference in the structure of amino acids that effectively modified the protein properties of the genes. Whereas there is a similar amino acid composition between the non-cyanidiophycean red algae (outgroup) and *C*-type. *G*-type mitogenomes have a distinct amino acid composition (Fig. 3a). *G*-type genomes show a higher proportion of positively charged amino acids than those of *C*-type (*G*-type: 10.77–10.82%, *C*-type: 6.84–7.44%; see in Fig. S6). A lower negative charge amino acid composition was found in *G*-type genomes when compared to *C*-type (*G*-type: 2.26–2.47%, *C*-type: 4.06–4.21%; see in Fig. S6). Likewise, the influence of amino acid changes altered protein charge, hydrophilicity, and hydrophobicity (Fig. 3b, c).

Hydrophilicity, which was measured for 16 conserved mitochondrial proteins (Fig. 3b), representing the *G*-type species showed relatively higher hydrophilic amino acids in mitochondrial proteins (50.56–50.97%) than those in other red algal species (*C*-type and outgroup species: 41.64–43.49%). Because some genes showed dramatic difference in amino acid composition or in protein size, we examined individual gene hydropathy (a scale of hydrophobicity and hydrophilicity) to avoid a biased assessment [47, 48]. *G*-type proteins are clearly less hydropathic than other groups (Fig. 3c) and 11/16 mitochondrial genes in *G*-type tend to have reduced protein length (Fig. S7A) when compared to other species. These dramatic differences in amino acids (e.g., charge, length) of mitochondrial genes are critical to protein structure that can affect solubility, stability, and their functions [49]. We applied in silico analysis (e.g., instability index, aliphatic index) to calculate the stability of conserved mitochondrial proteins and also found *G*-type and *C*-type have a few significant differences (Fig. S7B, C) in their mitochondrial proteins. The instability index estimates in vivo instability of proteins based on a comparison of dipeptides features between unstable proteins and stable proteins, whereas the aliphatic index is calculated using the relative volume of aliphatic side chains to estimated thermostability [50, 51]. Conserved mitochondrial genes in Cyanidiophyceae are mostly membrane-bound proteins or in mitochondria they form protein complexes (e.g., mitochondrial respiratory complexes). In these cases, protein folding, which is a key factor to understand protein stability and their activity, can be highly dependent on lipid composition of the mitochondrial membrane or a protein-protein interaction with other supermatrix-forming proteins [52, 53]. Therefore, these protein interactions with mitochondrial membrane lipids need further study.

### Extreme GC-skew in *Galdieria*-type mitogenomes and its associated characteristics

On the basis of mitogenomes comparison, *G*-type mitogenomes have distinctive characteristics, such as high gene divergence and asymmetric nucleotide substitution. The difference in GC-contents between the *C*-type and *G*-type is pervasive across genomes (Fig. S8) showing that *C*-type species have lower GC-contents (25.0–27.1%) than that of *G*-type species (41.4–44.0%) excluding mesophilic *Cd. chilense* (44.5%). However, GC-skew (G-C/G + C) and AT-skew (A-T/A + T) are clearly different in *C*-type and *G*-type (see Table 1): *C*-type composed symmetric AT and GC composition balances (AT-skew: 0.01–0.03; GC-skew: 0.01–0.06). In contrast, *G*-type showed unbalanced AT composition (AT-skew: 0.25–0.29) and extremely asymmetric composition of GC nucleotides (GC-skew: 0.66–0.74). Whereas in a member of the *C*-type species, mesophilic *Cd. chilense*, the GC-content (44.5%) is close to those of *G*-type, but GC-skew (0.01) or AT-skew (0.03) of mesophilic *Cd. chilense* are more similar to other *C*-type mitogenomes. In other words, mesophilic *Cd. chilense* may be regarded as an intermediate state between *C*-type and *G*-type based on its genomic features and phylogenetic position.

**Fig. 4:**
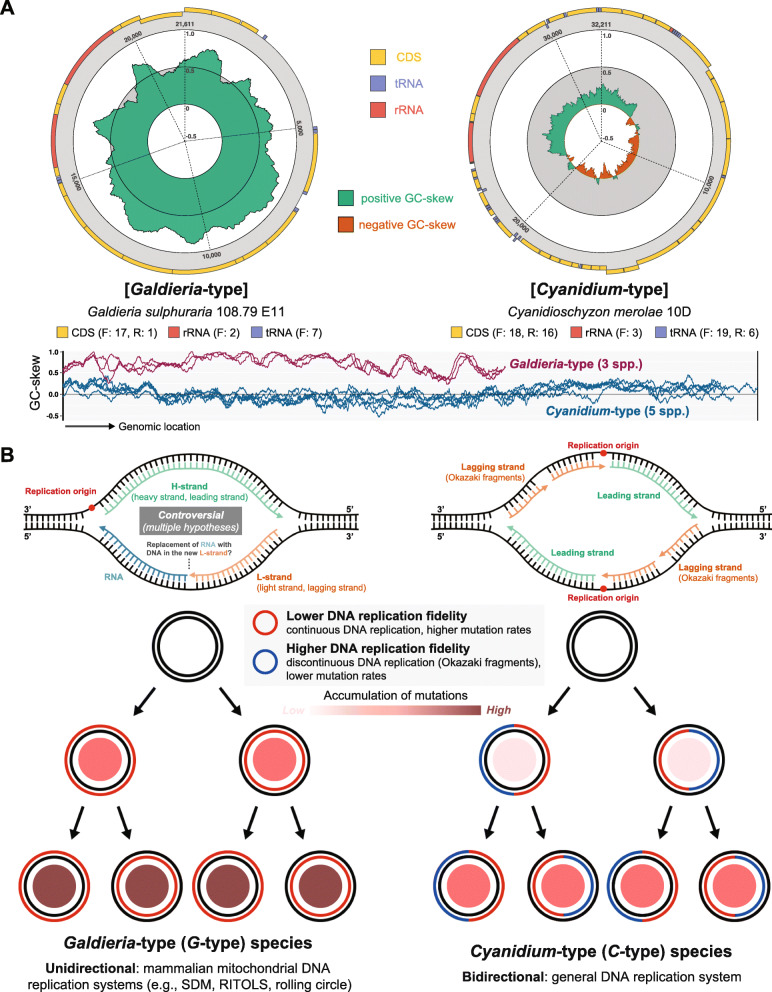
**Two different models for mitogenome replication in Cyanidiophyceae.** Unidirectional and conservative replication (separate leading and lagging strands for each daughter strand) in *Galdieria*-type and bidirectional and semiconservative replication (mixed leading and lagging strand for each daughter strand) in *Cyanidium*-type. **a** GC-skew of representative structure comparison. F: forward, R: reverse. **b** Hypothetical models of the mitochondrial DNA replication system and mitogenome inheritance model

All genes, including 17 CDSs, seven tRNAs, and two rRNAs, are located in a single strand of *G*-type *G. sulphuraria* 074 W mitogenome except for the anticlockwise *cob* gene, whereas genes in *C*-type *Cz. merolae* 10D mitogenome are distributed in both strands as usual (Fig. 4a). According to the directional distribution of genes and extreme GC-skew, genes in *G*-type mitogenome appear to be substantially strand-biased. The *cob* gene, which is located in an antisense orientation of *G*-type species, has lower GC-skew than average GC-skew of *G*-type mitogenomes (*cob* gene region GC-skew: 0.43–0.48, mitogenome GC-skew: 0.66–0.74) and shows a higher TIGER value compared to other genes meaning that *cob* gene contains lower variable sites (TIGER value of *cob* gene: 0.766, average TIGER value: 0.630; see Fig. S4, Table S2). Based on these observations, we examined the potential impact of extreme GC-skew on *G*-type mitogenomes.

One of the key approaches to distinguish leading and lagging strands is analysis of the GC-skew; a positive GC-skew reflects the leading strand, whereas a negative GC-skew represents the lagging strand in most cases [54, 55]. GC-skew analysis of the *G*-type shows all positive values and its cumulative GC-skew is gradually increased without any decreasing points unlike other red algal species, including *C*-type (Fig. 4a; Fig. S9). Mitogenomes with a positive GC-skew in a single strand have been well studied for their replication system, particularly in human mitochondria. Although DNA replication mechanisms in mitochondria are still under debate due to the absence of strong evidence for replication mode, unidirectional mitochondrial replication systems were reported in several mammalian mitochondria [56–65]. Furthermore, asymmetric DNA replication mechanism was reported in vertebrates based on the inversion of “control region (mtDNA non-coding fragment)”, which has strand-specific compositional bias [55, 66].

Unlike *C*-type species and other red algae, GC-skew analysis of *G*-type mitogenomes suggest that a leading strand and a lagging strand are separated into the individual strands (Fig. S9). Mitogenomes of *G*-type species have a guanine-rich leading strand (H-strand) and a cytosine-rich lagging strand (L-strand), thus, likely to have an asymmetric replication mechanism (Fig. 4). After the separation of L-strand from H-strand, without a bidirectional replication fork, a daughter lagging strand is continuously synthesized by a nascent leading strand and a nascent lagging strand synthesizes a daughter leading strand (Fig. 4b). The synthesis of lagging strand is considered to be more accurate than the leading strand due to lagging strand’s discontinuous replication (short Okazaki fragments) [61], which have been proposed in some mitochondria (e.g., yeast, fish) or bacterial species with experimental validation [67–71]. In addition, different gene substitution rates have been reported between two DNA strands of mitochondrial genome (e.g., higher in lagging strand of fish) [68, 72].

The mitogenome variants of *Cyanidiococcus yangmingshanensis* 8.1.23 F7 strain (*C*-type representative) and *Galdieria sulphuraria* 108.79 E11 strain (*G*-type representative), derived from a single cell, were verified to test replication-associated mutational stress. Illumina short-read data were used to identify SNPs in mitogenomes, and mapping coverages were 7931x for *Cc. yangmingshanensis* mitogenome and 7896x for *G. sulphuraria* mitogenome. Using GATK4 and Picard to call variants, we identified a single SNP from non-coding region of *Cc*. *yangmingshanensis* 8.1.23 F7 and four SNPs from non-coding region of *G. sulphuraria* 108.79 E11 (Table S3). Although the size of mitogenome is ~ 1.5x larger in *Cc*. *yangmingshanensis* 8.1.23 F7 than *G. sulphuraria* 108.79 E11, *G. sulphuraria* 108.79 E11 mitogenome (four SNPs) has more SNPs than *Cc*. *yangmingshanensis* 8.1.23 F7 mitogenome (a single SNP). More frequent mitogenome variants of *G*-type (0.031 SNPs/kbp) compared to *C*-type (0.185 SNPs/kbp) may explain the effect of replication-associated mutational stress [73]. Taken together, *G*-type species have higher mutation rates that are likely to be accelerated by unidirectional replication (Fig. 4b), which have also been proposed in the case of strand-the asymmetric model from inverted mitochondrial replication of vertebrate mitochondria [55].

On the basis of these findings, we propose unidirectional replication in *G*-type mitochondria that may have led to higher divergence than in the *C*-type and other non-cyanidiophycean red algae, which have bidirectional mitogenome replication. Because of mitochondrial replication system divergence, *G*-type mitogenomes may have different uses of the DNA polymerases described in Jain et al. [30], resulting in sequence variation including SNPs in *G*-type mitochondrial DNA. Such accelerated mutation rates of the two DNA strands may potentially contribute to *G*-type mitogenomes having a higher fitness in rapidly changing environments and play a role in adaptation [74]. We speculate that extreme GC-skew in the *G*-type resulted in the use of unidirectional replication that led to changes in protein properties, affecting the fitness of mitochondrial proteins in harsh environments.

## Conclusions

The rapid evolution we report here in *Galdieria*-type mitogenomes (i.e., long branches of *G*-type in the molecular phylogenies) may be explained by the fact that Cyanidiophyceae species inhabit extreme conditions unlike other mesophilic red algae. Among Cyanidiophyceae, *Cyanidium*-type (*C*-type) species inhabit ecologically more protected niches (e.g., aquatic habitats such as hot springs and ditches) within extreme environments than do *G*-type species. Therefore, they are likely to be less prone to environmental pressures such as temperature or pH fluctuation than *Galdieria* species [4, 10]. Reactive oxygen species in the internal environment of mitochondria, mitochondria replication, and absence or degeneration of mitochondrial DNA repair systems can elevate mitochondrial mutation [75]. In cyanidiophycean mitochondria, high temperature and an acidic environment are potential oxidative stress inducers, which are detrimental to cellular components like DNA or their repair system. Along with these common factors, another mutagenic factor could be the unidirectional replication mode of *G*-type mitogenomes. Strand-based compositional asymmetry of the two separate replicated strands can be introduced through replication-associated mutational stress [73]. However, in comparison to other eukaryotic mitogenomes that also have unidirectional replication, cyanidiophycean species have been exposed to extreme habitats and diverged into the *G*-type and *C*-type more than 800 Mya [13]. Since then, the unidirectional mitochondrial DNA replication system in *G*-type species has facilitated mutagenesis in stressful habitats over a long evolutionary time span, resulting in exceptionally divergent *G*-type mitochondria that is not observed in unidirectional replication systems of other red algae or even in nuclear and plastid of *G*-type species. Specifically, we assume that dominant unidirectional DNA replication was established in the ancestral population of *G*-type mitochondria. Unlike the nuclear genome, mitochondria are subject to rapid genetic drift due to predominantly uniparental inheritance and lack of sexual recombination [76]. Thus, in asexual species, the reduced effective population size of mitochondria, which weakens the power of natural selection (e.g., “*double rachet*” model) [76], may have allowed the initial survival of mutations that proved adaptive in the longer run, such as *G*-type mitochondrial DNA that confers thermostability of DNA and RNA (Supplementary Information 3).

However, because of their long evolutionary history, it is difficult, if not impossible, to assign a cause-and-effect relationship between a large mutagenesis event and unique trait evolution (e.g., protein properties). Interestingly, EGT-derived genes that were moved to the nucleus in the early stages of eukaryote evolution accumulated fewer mutations than mitochondrial-encoded genes (Supplementary Information 4). In addition, mutations of *G*-type mitochondrial genes are found to be under purifying selection, similar to mammalian mitochondrial genes [77], based on analysis of the nonsynonymous and synonymous mutation (K_a_/K_s_) ratio (Fig. S11). This suggests that *G*-type mitogenomes acquired mutations faster than other *C*-type mitogenomes or non-cyanidiophycean red algal mitogenomes during the same time period. The reason why these changes are restricted to the *G*-type mitochondrial genome and are not evident in plastid or nuclear DNA remains unclear. In summary, we speculate that a combination of factors (e.g., unidirectional replication system, polyextreme habitats, heterotrophic metabolism, reduced effective population size of mitochondria) have driven *G*-type mitogenome evolution. These results lay the foundation for future studies to better understand the biology of the intriguing Cyanidiophyceae.

## Methods

### Sample preparation

*Cyanidium caldarium* samples were obtained from the predecessor strain (*Cd. caldarium* ACUF 019; Siena, Italy) established by Claudia Ciniglia. Due to strain contamination issues in some isolated strains, single cells from two established strains of *Cyanidiococcus yangmingshanensis* (*Cc. yangmingshanensis* 8.1.23; Kula Manisa, Turkey) and *Galdieria sulphuraria* (*G. sulphuraria* SAG 108.79; Yellow Stone National Park, USA) were isolated using fluorescence-activated cell sorting (FACS) to establish single cell derived culture strains. After FACS isolation, and initial cultivation in a liquid medium (5x Allen medium), the cells were spread on a sucrose-agar medium, and an individual colony from each species (108.79: isolate E11, 8.1.23: isolate F7) was transferred to liquid media to promote growth. Blue-green biofilms were obtained from the shaded side of the Sybil Cave tuff wall (Cume, Italy) to collect mesophilic *Cyanidium chilense* (*Cd. chilense*). Collected samples were mixed with 15 mL phosphate-buffered saline (PBS) and centrifuged briefly at 550 rpm, repeatedly, to harvest cells from environmental samples.

### Transmission electron microscopy

Samples of *G. sulphuraria* 108.79 E11 and *Cc. yangmingshanensis* 8.1.23 F7 were initially fixed in a solution of 1.5% glutaraldehyde + 8% sucrose in 0.1 M phosphate buffer (pH 7.4) and incubated overnight at 4 °C. Fixed samples were rinsed for 10 min and centrifuged at 12,000 rpm for 5 min. After repeating the rinse three times, 1% osmium tetroxide in 0.1 M phosphate buffer was treated to the samples for 1.5 h. We repeated this step three times by rinsing the samples for 10 min and centrifuging at 12,000 rpm for 5 min. Pellets of fixed cyanidiophycean cells were dislodged and solidified in 1% agarose to divide them into the appropriate size for the section. The dehydration step in ethanol was done after the sectioning step and the pellet segments were embedded in Spurr’s resin. Samples were cut into 70 nm thickness and stained with uranyl acetate and lead citrate. The samples were observed using a Bio-HVEM System (JEM-1400 Plus at 100 kV and JEM-1000BEF at 1000 kV [JEOL, Japan]) at the Korea Basic Science Institute (Ochang, Korea).

### DNA extraction and *rbc*L sequencing

The genomic DNA of three culture strains (*Cd. caldarium* ACUF 019, *Cc. yangmingshanensis* 8.1.23 F7, *G. sulphuraria* 108.79 E11) was extracted by DNeasy Plant Mini Kit (Qiagen, Hilden, Germany) and purified using the LaboPass™ DNA Isolation Kit (Cosmo Genetech, Seoul, Korea). Genomic DNA of environmental samples from Sybil Cave was extracted using the FastDNA® SPIN Kit for Soil (MP Bio, Santa Ana, USA). To identify species, *rbc*L gene was amplified using DNA KOD FX Neo (Toyobo, Osaka, Japan) polymerase from each strain. A different combination of *rbc*L primer sets were used for PCR; *rbc*L_rc_214F: 5′-GTTGTWTGGACWGATTTATTAAC-3′ (23 mers), *rbc*L_rc_1234R: 5′-GCTTGWATWCCATCTGGATC-3′ (20 mers), *rbc*L_90F: 5′-CCATATGCYAAAATGGGATATTGG-3′ (24 mers), *rbc*L_R: 5′-ACATTTGCTGTTGGAGTCTC-3′ (20 mers) [78]. Amplified DNAs were purified by LaboPass™ PCR Purification Kit (COSMO Genetech, Seoul, Korea) and products were sent to a sequencing company (Macrogen, Seoul, Korea). We manually removed low quality sequences and merged forward and reverse strand sequences into a single aligned sequence.

### Whole genome sequencing and mitogenome analysis

The raw data of *Galdieria phlegrea* DBV 009 (Naples, Italy), sequenced in a previous study [27], was used here to complete the mitogenome. Other whole genome data were generated using the Illumina HiSeq 2500 (Illumina, San Diego, USA) with 2 × 100 bp paired-end sequencing at the DNA Link (Seoul, Korea). Reads were assembled into contigs using SPAdes assembler v3.10.1 [79] and mitogenomes were constructed following the established procedure described in Cho et al., 2018 [80] using two published mitogenomes (NC_000887, NC_024666) as reference. Using the mitochondrial proteins of *Cz. merolae* 10D [31], assembled contigs that include mitochondrial genes were selected by tBLASTn. Next, the sorted contigs were re-assembled to get a scaffold sequence of mitogenomes using Geneious Prime 2019.0.3 (Biomatters, Auckland, New Zealand). After the re-assembly step, a complete circular mitogenome or partial linear mitogenome contigs were obtained from each species. The completeness of these mitogenome sequences were tested by LASTZ [81] and by comparison with two published mitogenomes (NC_000887: *Cz. merolae* 10D, NC_024666: *G. sulphuraria* 074 W). The genome sequences were mapped with raw reads to correct sequencing errors by mapping coverage or filling gaps for a linear mitogenome.

Protein coding genes (CDS) were manually annotated based on a homologous region of existing mitogenomes (NC_000887, NC_024666, KJ569774). The “standard genetic code 1” has been generally used in cyanidiophycean mitogenomes [30, 31] without clear justification, although there is a report of using “protozoan mitochondrial genetic code 4” in the Gigartinales (red algae) [82]. To test which genetic code is better suited to our data, we translated the genes using both genetic codes, “standard genetic code 1” and “protozoan mitochondrial genetic code 4”. Because the “protozoan mitochondrial genetic code 4” resulted a 5′-extension that resulted in a gene annotation error (see Fig. S12), we chose to use the “standard genetic code 1” in this study. The ribosomal RNA sequences were acquired by BLASTn searching against other mitochondrial rDNA sequences of Cyanidiophyceae. tRNAs, and other small RNAs were searched by using tRNAscan-SE 2.0 [83] and ARAGORN [84]. For comparative analysis using mitochondrial genes, several genes and ambiguous sequences from the published mitogenomes were re-annotated (Table S5) in this study [85]. GC-content was calculated with a sliding window scale of 48 bp, and the GC-skew of each species was calculated (window size: 1 kbp, step: 20 bp) using a Python script. Potential G-quadruplex forming sequences were predicted by G4Hunter using a default option [86].

### Phylogenetic analyses

To identify the phylogenetic relationship of the newly sequenced strains among the taxon-rich *rbc*L phylogeny, all available *rbc*L sequences of Cyanidiophyceae were collected from the NCBI nucleotide database (ntDB). After discarding the duplicated data, a total of 269 *rbc*L sequences including the five generated here was used for phylogenetic analysis with 10 other red algal species (i.e., Rhodophytina) as outgroups. For phylogenetic analysis of mitochondrial genes, eight Cyanidiophyceae taxa were selected, along with four other red algae as outgroups. The outgroup taxa included two species from Florideophyceae (*Chondrus crispus* and *Hildenbrandia rubra*), one species from Bangiophyceae (*Porphyra purpurea*), and one species from Compsopogonophyceae (*Compsopogon caeruleus*). A total of 32 mitochondrial proteins presents in at least five species out of eight cyanidiophycean species were concatenated for the ML analysis. Gene alignments were produced using MAFFT v7.310 [87] with the default options. Maximum likelihood (ML) phylogenetic analysis with automated model selection was done using IQ-TREE v1.6.8 [88] with 1000 ultrafast bootstrap replications. The model test option in IQ-TREE was applied to each protein in this alignment.

### Comparison of mitochondrial genes

Because most of datasets used for statistical tests did not show a normal distribution, the ‘Independent-Samples Kruskal-Wallis Test’ was used to evaluate hypotheses (Table S6). The null hypothesis was that the distribution of factor (e.g., amino acid composition) is identical among taxa from two ingroup clades (i.e., *Cyanidium*-type and *Galdieria*-type) and outgroup taxa. If the null hypothesis is rejected, pairwise comparisons among three groups were applied (Table S7). Sixteen conserved mitochondrial genes (*sdh*C, *atp*4, *atp*6, *atp*8, *atp*9, *cob*, *cox*1, *cox*2, *cox*3, *nad*1, *nad*2, *nad*3, *nad*4, *nad*4L, *nad*5, *nad*6) that all encoded in mitogenomes of 13 species were chosen to test amino acid properties.

The alignment of each protein and their ML trees were used for pairwise comparisons and all results were summarized in Table S8. Only conserved sites excluding any gaps were selected from the protein alignment of conserved genes for analysis (3845/4825 sites). Average proportions of amino acids were recorded in each group and the values were visualized with standard errors.

The GRAVY value, which is determined by the total hydropathic values of all amino acids in the protein divided by its length, is used to represent its hydrophobic or hydrophilic properties for analysis. To estimate protein stability, two different types of indices were used in this study. The aliphatic index indicating thermostability of the globular protein is calculated by the relative volume of aliphatic side chains in the protein [50], and the instability index provides an estimate of in vivo protein stability based on protein dipeptide differences [51]. To compare protein properties for 16 conserved mitochondrial genes, GRAVY (grand average of hydropathy), aliphatic index, and instability index were calculated using ProtParam in ExPASy [48], and the results were summarized in Table S9. The mean values of each feature were illustrated with bar plots with standard error bars.

For the EGT-derived gene detection and transit peptide prediction, nuclear genomes of *G. sulphuraria* 074 W and *Cz. merolae* 10D were used as references to find mitochondrion-derived nuclear-encoded genes [28, 39, 89]. EGT genes were further verified based on homologous search by MMSeqs2 [90]. Transit peptide sequences in EGT candidates were predicted by TargetP [91].

### Mitogenome variant calling

Due to sequences of mixed species (*Cyanidium caldarium*) from the published genome data of *Galdieria phlegrea* DBV 009 [27], we only chose two FACS-derived strains (*Cyanidiococcus yangmingshanensis* 8.1.23 F7 and *Galdieria sulphuraria* 108.79 E11) to identify single nucleotide polymorphisms (SNPs). Bowtie2 v.2.3.5.1 [92] was used to map the sequencing data to each mitogenome (Table S3). Based on these mapping information, we used Picard v1.4.2 (see http://broadinstitute.github.io/picard/) and GATK4 v4.1.2.0 [93] to identify SNPs.

### Estimation of evolutionary rate and analysis of selection

Concatenated datasets of mitochondrial proteins were divided into 64 separate partitions (‘-b 100’: allocate the sites in 1 to 100 based on the evolutionary rates) based on TIGER values (Tables S1, S2), which calculate the evolutionary rates for each site on the basis of tree-independent approaches to differentially weighted characters [94]. Non-synonymous substitutions per non-synonymous sites (K_a_) and synonymous substitutions per synonymous sites (K_s_) have been widely used to determine evolutionary selection on genes [95]. A total of 16 conserved mitochondrial genes were selected for alignments to calculate ratios of non-synonymous substitutions per non-synonymous sites and synonymous substitutions per synonymous sites (K_a_/K_s_), which have been widely used to determine the evolutionary selection on genes [95]. ParaAT [96] was used with MAFFT version 7 [87] and KaKs_Calculator [97] to do this analysis.

## Supplementary information

**Additional file 1 Electronic Supplementary Materials.** Supplementary materials (pdf format), contain Supplementary Information 1–4, Supplementary Figures. S1-S12, and Supplementary References (references that were used in Supplementary Information 1–4). Supplementary Information 1–4: additional information describing the results of this study but not the necessary information to be provided in the main text. Supplementary Figures. S1-S13: additional data with legends to support the manuscript.

**Additional file 2 Supplementary Tables S1-S9.** Supplementary materials (xlsx format), contain Supplementary Tables S1-S9. Supplementary Tables S1-S9: additional data with legends to support the manuscript.

## Data Availability

The mitogenome sequences have been deposited at GenBank under the accession numbers MT270115-MT270119. All sequence alignments and data are available from Dryad Digital Repository (10.5061/dryad.nvx0k6dp7).

## Abbreviations

*C*-type: *Cyanidium*-type
*G*-type: *Galdieria*-type
CZME: *Cyanidioschyzon merolae*
CYSP: Cyanidiophyceae sp.
CCYA: *Cyanidiococcus yangmingshanensis*
CDCA: *Cyanidium caldarium*
CDCH: *Cyanidium chilense*
GAPH: *Galdieria phlegrea*
GASU: *Galdieria sulphuraria*
