# Correction to: Potential causes and consequences of rapid mitochondrial genome evolution in thermoacidophilic Galdieria (Rhodophyta)

**DOI:** 10.1186/s12862-020-01686-5

**Published:** 2020-10-07

**Authors:** Chung Hyun Cho, Seung In Park, Claudia Ciniglia, Eun Chan Yang, Louis Graf, Debashish Bhattacharya, Hwan Su Yoon

**Affiliations:** 1 Department of Biological Sciences, Sungkyunkwan University, Suwon, 16419 South Korea; 2 Department of Environmental, Biological and Pharmaceutical Science and Technologies, University of Campania Luigi Vanvitelli, 81100 Caserta, Italy; 3 Marine Ecosystem Research Center, Korea Institute of Ocean Science and Technology, Busan, 49111 South Korea; 4 Department of Biochemistry and Microbiology, Rutgers University, New Brunswick, 08901 USA

**Correction to: BMC Evolutionary Biology 20, 112 (2020)**

**https://doi.org/10.1186/s12862-020-01677-6**

Following publication of the original article [1], the authors identified an error in Fig. 1. and Fig. 4. The correct figures are given below.

**Fig. 1:**
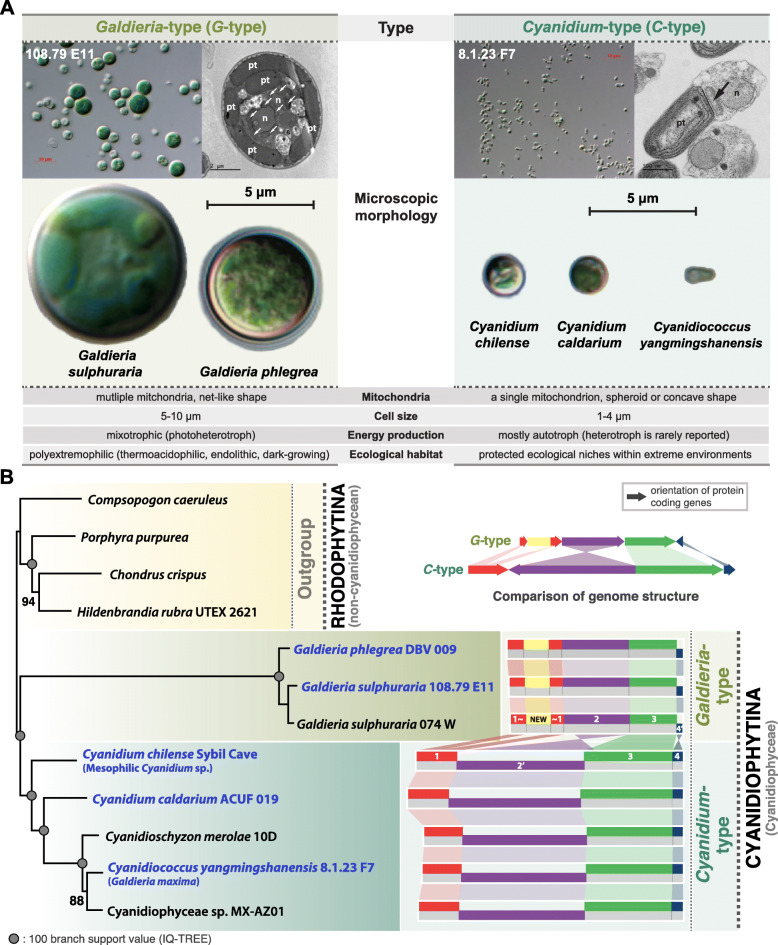
Overview of the major characteristics of Cyanidiophyceae and its phylogeny. **a** Comparison of key characteristics of the *Cyanidium*-type and *Galdieria*-type species showing two different types of cyanidiophycean cells. Based on existing studies, key characteristics were summarized in this figure with n: nucleus, pt: plastid, and arrow: mitochondria. **b** Maximum-likelihood phylogeny using a concatenated 32-protein alignment of 12 mitochondrial genomes. Four non-cyanidiophycean species were chosen as the outgroup. The simplified genome structure of cyanidiophycean mitochondria is illustrated next to the phylogenetic tree

**Fig. 4:**
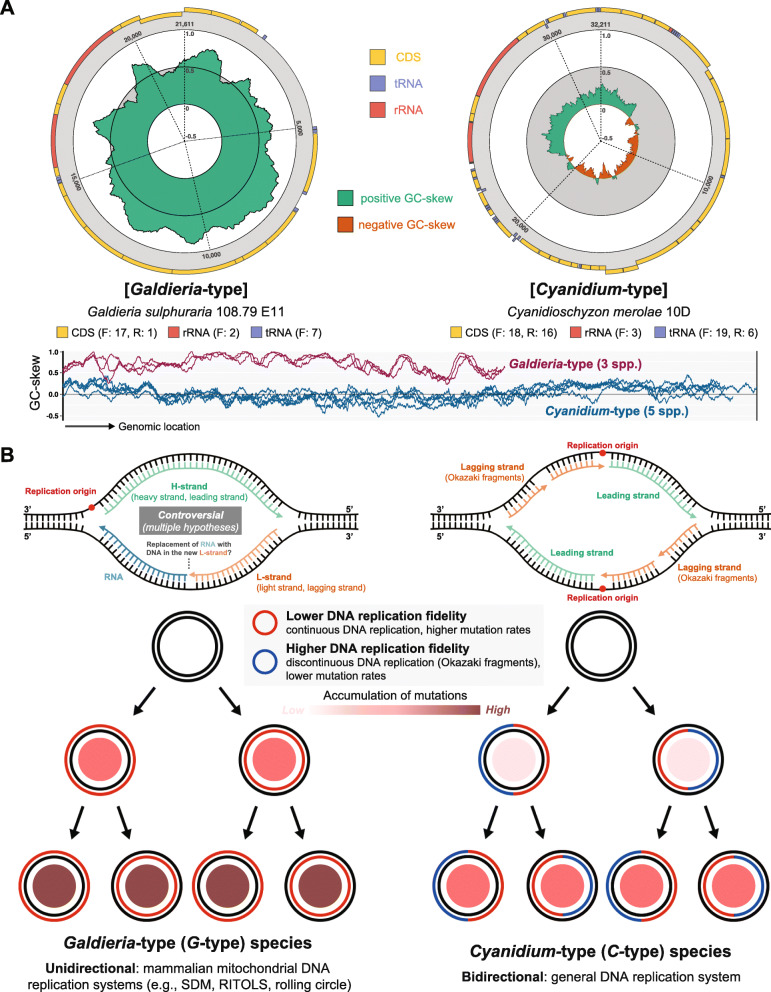
Two different models for mitogenome replication in Cyanidiophyceae. Unidirectional and conservative replication (separate leading and lagging strands for each daughter strand) in *Galdieria*-type and bidirectional and semiconservative replication (mixed leading and lagging strand for each daughter strand) in *Cyanidium*-type. **a** GC-skew of representative structure comparison. F: forward, R: reverse. **b** Hypothetical models of the mitochondrial DNA replication system and mitogenome inheritance model

The correct figures and captions have been included in this correction, and the original article has been corrected.
