# Supplementary material for: Potential causes and consequences of rapid mitochondrial genome evolution in thermoacidophilic Galdieria (Rhodophyta)
Source: BMC Evol Biol. 2020 Sep 7;20:112. doi: 10.1186/s12862-020-01677-6 (PMC7487498; doi:10.1186/s12862-020-01677-6)
Supplement: Supplementary file 1 — Additional file 1 Electronic Supplementary Materials. Supplementary materials (pdf format), contain Supplementary Information 1–4, Supplementary Figures. S1-S12, and Supplementary References (references that were used in Supplementary Information 1–4). Supplementary Information 1–4: additional information describing the results of this study but not the necessary information to be provided in the main text. Supplementary Figures. S1-S13: additional data with legends to support the manuscript. [file 12862_2020_1677_MOESM1_ESM.pdf]

Electronic Supplementary Materials

for

Potential causes and consequences of rapid mitochondrial genome evolution in thermoacidophilic *Galdieria* (Rhodophyta)

Chung Hyun Cho[1†], Seung In Park[1†], Claudia Ciniglia[2], Eun Chan Yang[3], Louis Graf[1], Debashish Bhattacharya[4], Hwan Su Yoon[1*]

[1]Department of Biological Sciences, Sungkyunkwan University, Suwon 16419, Korea, [2]Department of Environmental, Biological and Pharmaceutical Science and Technologies, University of Campania Luigi Vanvitelli, Caserta 81100, Italy, [3]Marine Ecosystem Research Center, Korea Institute of Ocean Science and Technology, Busan 49111, Korea, [4]Department of Biochemistry and Microbiology, Rutgers University, New Brunswick 08901, USA

[*]Author for correspondence: Hwan Su Yoon, e-mail: hsyoon2011@skku.edu

[†]These authors contributed equally to this work.

Electronic Supplementary Materials:

Supplementary Information 1-4

Supplementary References

Supplementary Figures S1-S12

Supplementary Tables S1-S9

Supplementary Information 1-4:

Supplementary Information 1. Individual CDS phylogenies and evolutionary rates

Tree topologies are inconsistent between individual gene trees and the concatenated gene tree. Thirty-two mitochondrial genes (also see Figure S3 for different taxon numbers for each analysis), which are present in *C*-type mitogenomes, were used to compare the phylogenetic relationships of each group of i) the *G*-type (nodes *b* and *c*), ii) the *C*-type (nodes *d*-*g*), and iii) the Cyanidiophyceae (node *a*; *G*-type + *C*-type). All 18 mitochondrial genes (18/18: 100%) in *G*-type genomes supported the monophyly of three *Galdieria* taxa (node b), but the monophyly of *G. sulphuraria* strains (node *c*) consistent in 12 genes (12/18: 66.7%) (Figure S3). Monophyly of the *C*-type (node *d*) was supported by 17 out of 26 genes (17/26: 65.4%), and only two out of 17 genes (2/17: 11.8%) supported their internal relationship (nodes *e*-*g*; Figure S3). Lastly, the monophyly of Cyanidiophyceae (node *a*) was supported by 10/22 (45.5%) mitochondrial genes without considering internal relationships (Figure S3). Overall, only two mitochondrial genes, *cob* and *cox*1, supported the (potential) species tree relationship of Cyanidiophyceae (Figure S3). These two genes are widely used to identify species delimitation as MT barcoding markers [1-5].

The evolutionary rates of each individual gene were calculated using TIGER (Figure S4, Tables S1, S2). A higher TIGER value and a lower Bin number indicates more conservation. TIGER evolutionary rate analysis identified that *sdh*D (0.49) showed the lowest value and *atp*9 (0.90) the highest value, with an average of 0.63 for all sites (Figure S4, Table S2). Other gene families (gene family that consists at least two genes), all mitochondrial-encoded cytochrome *c* oxidase (*cox*1-3*,* mitochondria complex IV) genes have higher TIGER values than the average, implying that *cox* genes have evolved more slowly. Interestingly, two mitochondrial genes (*cob* and *cox*1) represent high TIGER values (2nd, 3rd highest TIGER value), and individual tree topologies were congruent with the concatenation tree. On the other hand, although the *atp*9 gene has the highest TIGER value, it could not resolve internal relationships of each group likely due to its short protein length (76 amino acids).

Supplementary Information 2. A unique horizontal gene uptake of a prokaryotic *ccm*F gene into *Galdieria*-type mitogenome

Several genome studies have suggested that cyanidiophycean species acquired bacterial or archaeal genes to adapt to extreme conditions [6-8]. The horizontal gene transfer of the *ccm*F (cytochrome *c* biogenesis protein CcmF) gene, which forms a complex of heme lyase to transfer heme from CcmE to apocytochrome *c* [9], in *G*-

type mitochondria has likely replaced non-α-proteobacterium-derived *ccm*F genes (Figure S5). Typical α-proteobacterium-derived mitochondrial *ccm*F of *C*-type and other eukaryotes form clusters, *G*-type *ccm*F showed monophyly (91% MLB) with other prokaryotic *ccm*F (e.g., Archaea and some bacterial lineages). This supports the idea that prokaryote gene transfers with replacement of existing gene may have contributed to adaptation to extreme environments [7].

Supplementary Information 3. Impacts of GC skewness in mitogenomes: potential G-quadruplex sequences (pG4s)

Potential G-quadruplex sequences (pG4s), which are candidates to form four-stranded helical structure driven by the self-association of guanines [10], results in more complex DNA or RNA structures [11]. Forming G-quadruplex DNA structures using ions (e.g., K^+), G-quadruplex-forming ssDNA is more stable than dsDNA (20–30°C higher melting temperatures), indicating specific proteins are needed to unfold them into a linear form [12]. Not only are G-quadruplexes related to DNA stability, they are also involved in multiple biological processes, such as DNA replication, transcription, translation, and epigenetic regulation [13]. For example, by physically blocking replication or transcription, the pG4s-induced folded DNA structure can regulate metabolic pathways [14]. Similarly, it was proposed that GC skewness promotes a potential increase in mitogenomic G-quadruplex formation [15], therefore we compared the numbers of pG4s between the two types of mitogenomes based on high GC skew in *G*-type mitogenomes. *G*-type mitogenomes contain higher numbers of pG4s (42-73; 1.9-2.4 bp per 1 kbp) than those of *C*-type (2-9; 0.1-0.3 bp per 1 kbp), however there was no clear distributional bias (e.g., pG4s-rich in coding or non-coding regions) and a random distribution of these regions in *G*-type mitogenomes (Figure S10). Guanine-rich mRNAs may promote transcript stability to prevent degradation in heat stress [16], therefore, extreme GC skew in *G*-type species may be an adaptation to extreme conditions.

Supplementary Information 4. Evolution of ancient nuclear-encoded mitochondrial genes

The protein properties of *G*-type mitochondrial genes, as previously described, clearly represent higher accumulation of mutations than in *C*-type. To test whether nuclear-encoded mitochondrial genes (ancient EGT) have also evolved rapidly, we surveyed six representative nuclear-encoded mitochondrial genes (*CYT1*, *NDUFS1, NDUFS2, NDUFA2, ISP, QCR9*) involved in oxidative phosphorylation (KEGG pathway: ko00190) from three accessible nuclear genomes (i.e., *C*-type: *Cyanidioschyzon merolae* 10D, *G*-type: *Galdieria sulphuraria* 074W, outgroup red algae: *Chondrus cripsus*). Unlike mitochondrial-encoded genes, no significant

differences were observed from ancient mitochondrial EGT (Table S4). This suggests that selection pressure may have existed on mitogenome-encoded genes but not on nuclear-encoded, anciently derived EGT genes.

Supplementary References:

1. Robba L, Russell SJ, Barker GL, Brodie J: **Assessing the use of the mitochondrial *cox*1 marker for use in DNA barcoding of red algae (Rhodophyta)**. *Am J Bot* 2006, **93**(8):1101-1108.
2. Yang EC, Kim MS, Geraldino PJL, Sahoo D, Shin J-A, Boo SM: **Mitochondrial *cox*1 and plastid *rbc*L genes of *Gracilaria vermiculophylla* (Gracilariaceae, Rhodophyta)**. *J Appl Phycol* 2008, **20**(2):161-168.
3. Kim KM, Hoarau GG, Boo SM: **Genetic structure and distribution of *Gelidium elegans* (Gelidiales, Rhodophyta) in Korea based on mitochondrial *cox*1 sequence data**. *Aquat Bot* 2012, **98**(1):27-33.
4. Saunders GW, Moore TE: **Refinements for the amplification and sequencing of red algal DNA barcode and RedToL phylogenetic markers: a summary of current primers, profiles and strategies**. *Algae* 2013, **28**(1):31-43.
5. Lam DW, Verbruggen H, Saunders GW, Vis ML: **Multigene phylogeny of the red algal subclass Nemaliophycidae**. *Mol Phylogenetics Evol* 2016, **94**:730-736.
6. Qiu H, Price DC, Weber APM, Reeb V, Chan Yang E, Lee JM, Kim SY, Yoon HS, Bhattacharya D: **Adaptation through horizontal gene transfer in the cryptoendolithic red alga *Galdieria phleagrea***. *Curr Biol* 2013, **23**(19):R865-R866.
7. Schönknecht G, Chen W-H, Ternes CM, Barbier GG, Shrestha RP, Stanke M, Bräutigam A, Baker BJ, Banfield JF, Garavito RM: **Gene transfer from bacteria and archaea facilitated evolution of an extremophilic eukaryote**. *Science* 2013, **339**(6124):1207-1210.
8. Rossoni A, Price D, Seger M, Lyska D, Lammers P, Bhattacharya D, Weber A: **The genomes of polyextremophilic Cyanidiales contain 1% horizontally transferred genes with diverse adaptive functions**. *eLife* 2019, **8**.
9. Ren Q, Ahuja U, Thöny-Meyer L: **A bacterial cytochrome *c* heme lyase CcmF forms a complex with the heme chaperone CcmE and CcmH but not with apocytochrome *c***. *J Biol Chem* 2002, **277**(10):7657-7663.
10. Rhodes D, Lipps H: **G-quadruplexes and their regulatory roles in biology**. *Nucleic Acids Res* 2015, **43**(18):8627-8637.

11. Bochman ML, Paeschke K, Zakian VA: **DNA secondary structures: stability and function of G-quadruplex structures**. *Nature Rev Genet* 2012, **13**:770.

12. Lipps HJ, Rhodes D: **G-quadruplex structures: *in vivo* evidence and function**. *Trends Cell Biol* 2009, **19**(8):414-422.

13. Puig Lombardi E, Holmes A, Verga D, Teulade-Fichou M-P, Nicolas A, Londoño-Vallejo A: **Thermodynamically stable and genetically unstable G-quadruplexes are depleted in genomes across species**. *Nucleic Acids Res* 2019, **47**(12):6098-6113.

14. Mendoza O, Bourdoncle A, Boulé J-B, Brosh RM, Jr, Mergny J-L: **G-quadruplexes and helicases**. *Nucleic Acids Res* 2016, **44**(5):1989-2006.

15. Pietras Z, Wojcik MA, Borowski LS, Szewczyk M, Kulinski TM, Cysewski D, Stepien PP, Dziembowski A, Szczesny R: **Dedicated surveillance mechanism controls G-quadruplex forming non-coding RNAs in human mitochondria**. *Nat Commun* 2018, **9**(1):2558.

16. Jain K, Krause K, Grewe F, Nelson GF, Weber APM, Christensen AC, Mower JP: **Extreme features of the *Galdieria sulphuraria* organellar genomes: a consequence of polyextremophily?** *Genome Biol Evol* 2015, **7**(1):367-380.

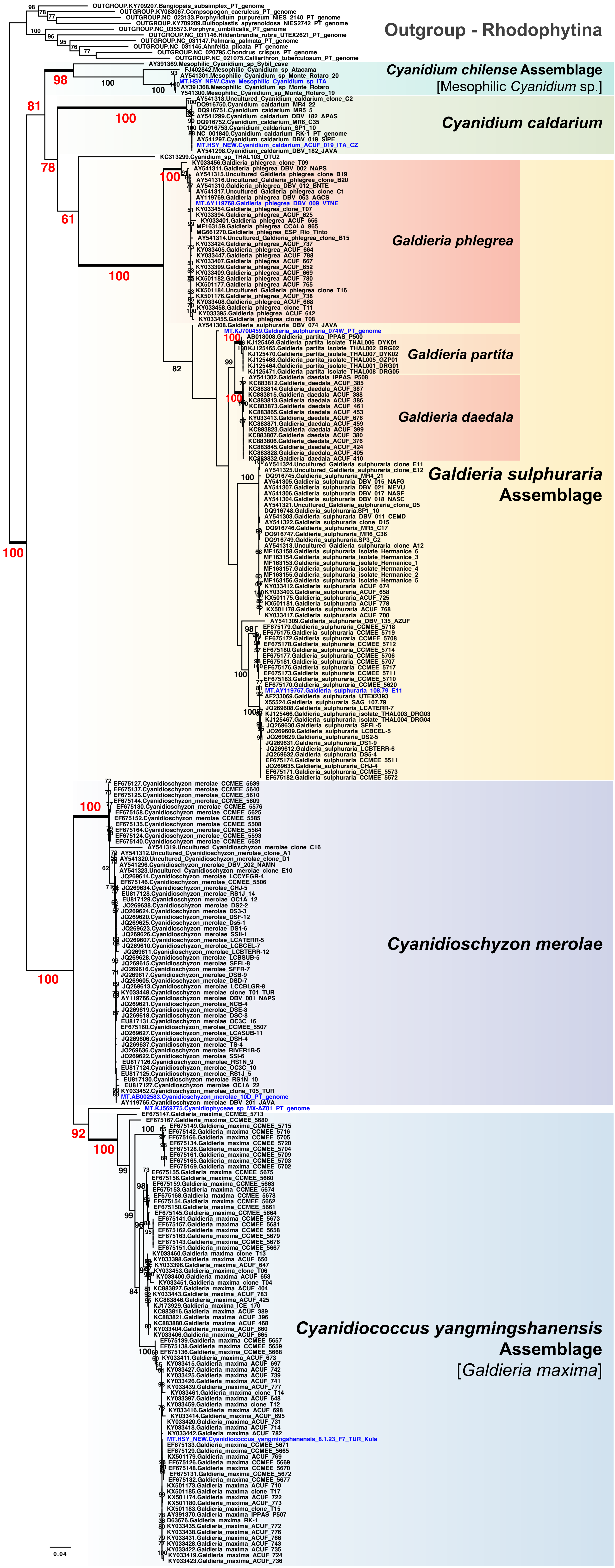

Supplementary Figure S1. Phylogeny of the Cyanidiophyceae inferred from a maximum likelihood analysis of plastidial *rbc*L gene sequences. The NCBI accession number of each sequences is shown preceding each species name. The strains that were used in the mitogenome study are marked in blue.

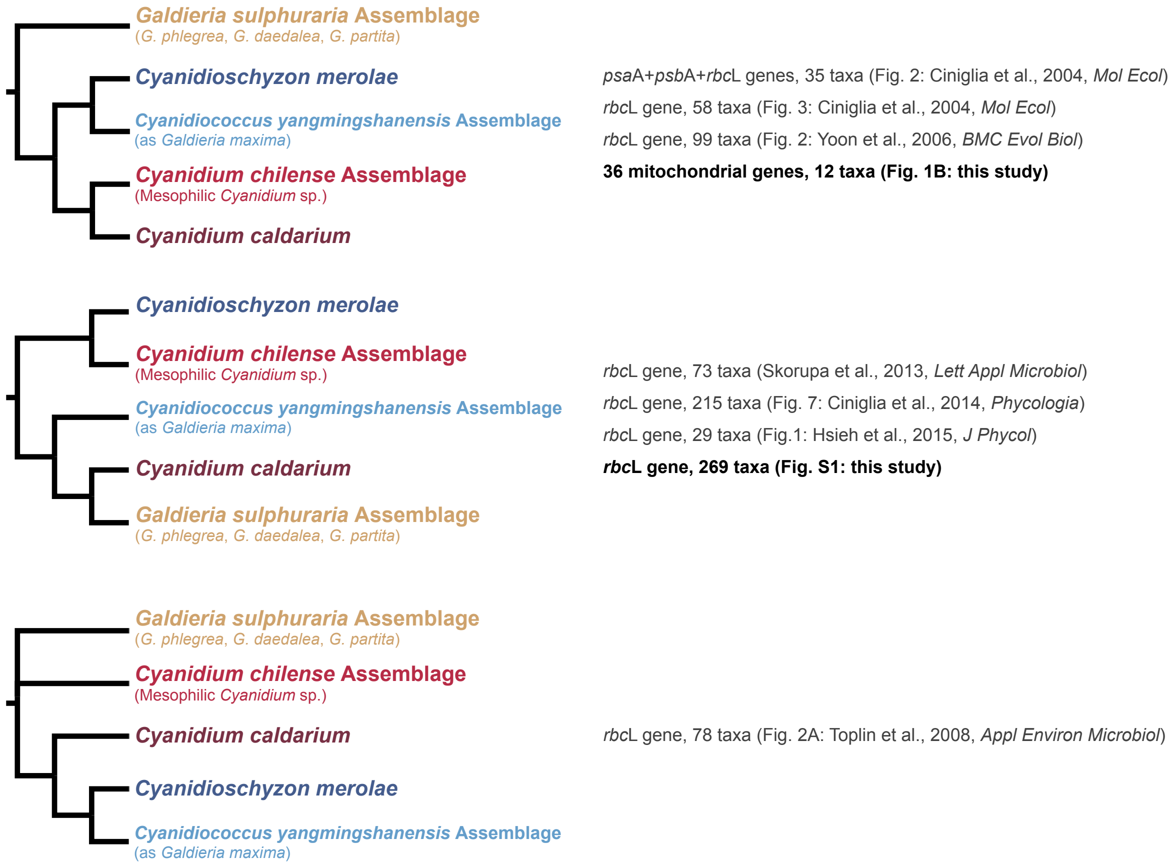

Supplementary Figure S2. Controversial evolutionary relationships of the Cyanidiophyceae from previous phylogenetic studies. Previous studies not involving five representative clades are excluded from this figure.

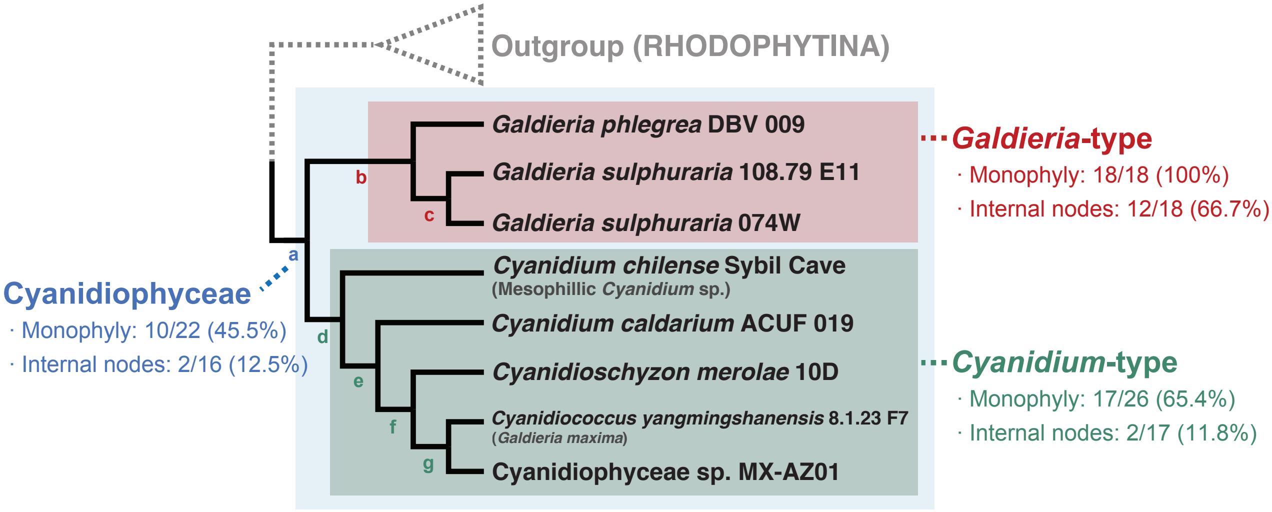

Legend: supported (support value ≥ 50) (green); not supported (red)

	Gene (no. of spp.)	Cyanidiophyceae: Monophyly	Cyanidiophyceae: Support Internal Nodes	*Galdieria*-type (*G*-type): *G*-type Monophyly	*Galdieria*-type (*G*-type): Support *G*-type Internal Nodes	*Cyanidium*-type (*C*-type): *C*-type Monophyly	*Cyanidium*-type (*C*-type): Support *C*-type Internal Nodes
ATP Synthase (Complex V)	atp4 (12 spp.)	X (paraphyly)	X	O	X	X	N/A (C-type paraphyly)
	atp6 (12 spp.)	X (39)	X	O	X	O	X
	atp8 (12 spp.)	X (paraphyly)	X	O	O	X	N/A (C-type paraphyly)
	atp9 (12 spp.)	O	X	O	X	O	X
Cytochrome *c* Biogenesis	ccmA (5 spp.)	N/A (no outgroup)	N/A (missing taxa)	N/A (gene absent)	N/A (gene absent)	N/A (no outgroup)	N/A (no outgroup)
	ccmB (5 spp.)	N/A (no outgroup)	N/A (missing taxa)	N/A (gene absent)	N/A (gene absent)	N/A (no outgroup)	N/A (no outgroup)
	ccmC (8 spp.)	N/A (no outgroup)	N/A (no outgroup)	O	O	O	X
	ccmF (8 spp.)	N/A (no outgroup)	N/A (no outgroup)	O	X	O	X
**Cytochrome *bc*$_1$** (Complex III)	cob (12 spp.)	O	O	O	O	O	O
Cytochrome *c* Oxidase (Complex IV)	cox1 (12 spp.)	O	O	O	O	O	O
	cox2 (12 spp.)	X (paraphyly)	X	O	O	X	N/A (C-type paraphyly)
	cox3 (12 spp.)	X (paraphyly)	X	O	O	X	N/A (C-type paraphyly)
NADH Dehydrogenase (Complex I)	nad1 (12 spp.)	O	X	O	O	X	N/A (C-type paraphyly)
	nad2 (12 spp.)	X (paraphyly)	X	O	X	X	N/A (C-type paraphyly)
	nad3 (12 spp.)	O	X	O	X	X	N/A (C-type paraphyly)
	nad4 (12 spp.)	X (paraphyly)	X	O	O	O	X
	nad4L (12 spp.)	X (paraphyly)	X	O	O	O	X
	nad5 (12 spp.)	X (paraphyly)	X	O	O	O	X
	nad6 (12 spp.)	X (paraphyly)	X	O	O	O	X
Ribosomal Proteins	rpl5 (5 spp.)	N/A (no outgroup)	N/A (missing taxa)	N/A (gene absent)	N/A (gene absent)	N/A (no outgroup)	N/A (no outgroup)
	rpl14 (5 spp.)	N/A (no outgroup)	N/A (missing taxa)	N/A (gene absent)	N/A (gene absent)	N/A (no outgroup)	N/A (no outgroup)
	rpl16 (8 spp.)	O (G-type absent)	N/A (missing taxa)	N/A (gene absent)	N/A (gene absent)	O	X
	rpl20 (7 spp.)	X (paraphyly)	N/A (missing taxa)	N/A (gene absent)	N/A (gene absent)	X	N/A (C-type paraphyly)
	rps3 (9 spp.)	O (G-type absent)	N/A (missing taxa)	N/A (gene absent)	N/A (gene absent)	O	X
	rps4 (5 spp.)	N/A (no outgroup)	N/A (missing taxa)	N/A (gene absent)	N/A (gene absent)	N/A (no outgroup)	N/A (no outgroup)
	rps11 (8 spp.)	O (G-type absent)	N/A (missing taxa)	N/A (gene absent)	N/A (gene absent)	O	X
	rps12 (8 spp.)	O (G-type absent)	N/A (missing taxa)	N/A (gene absent)	N/A (gene absent)	O	X
	rps14 (5 spp.)	N/A (no outgroup)	N/A (missing taxa)	N/A (gene absent)	N/A (gene absent)	N/A (no outgroup)	N/A (no outgroup)
Succinate Dehydrogenase (Complex II)	sdhB (8 spp.)	N/A (no outgroup)	N/A (missing taxa)	N/A (gene absent)	N/A (gene absent)	O	X
	sdhC (12 spp.)	X (paraphyly)	X	O	O	O	X
	sdhD (8 spp.)	X (paraphyly)	N/A (missing taxa)	N/A (gene absent)	N/A (gene absent)	X	N/A (C-type paraphyly)
Protein Translocase	tatC (9 spp.)	O (G-type absent)	N/A (missing taxa)	N/A (gene absent)	N/A (gene absent)	O	X

Supplementary Figure S3. Comparison of concatenated data and individual gene trees. The monophyletic origin of each group (*C*-type, *G*-type, Cyanidiophyceae) was studied. Bootstraps values under 50 were not considered in this analysis. The concordance of branching patterns between individual and concatenated gene trees are marked in different colors (green: concordance, red: incongruence).

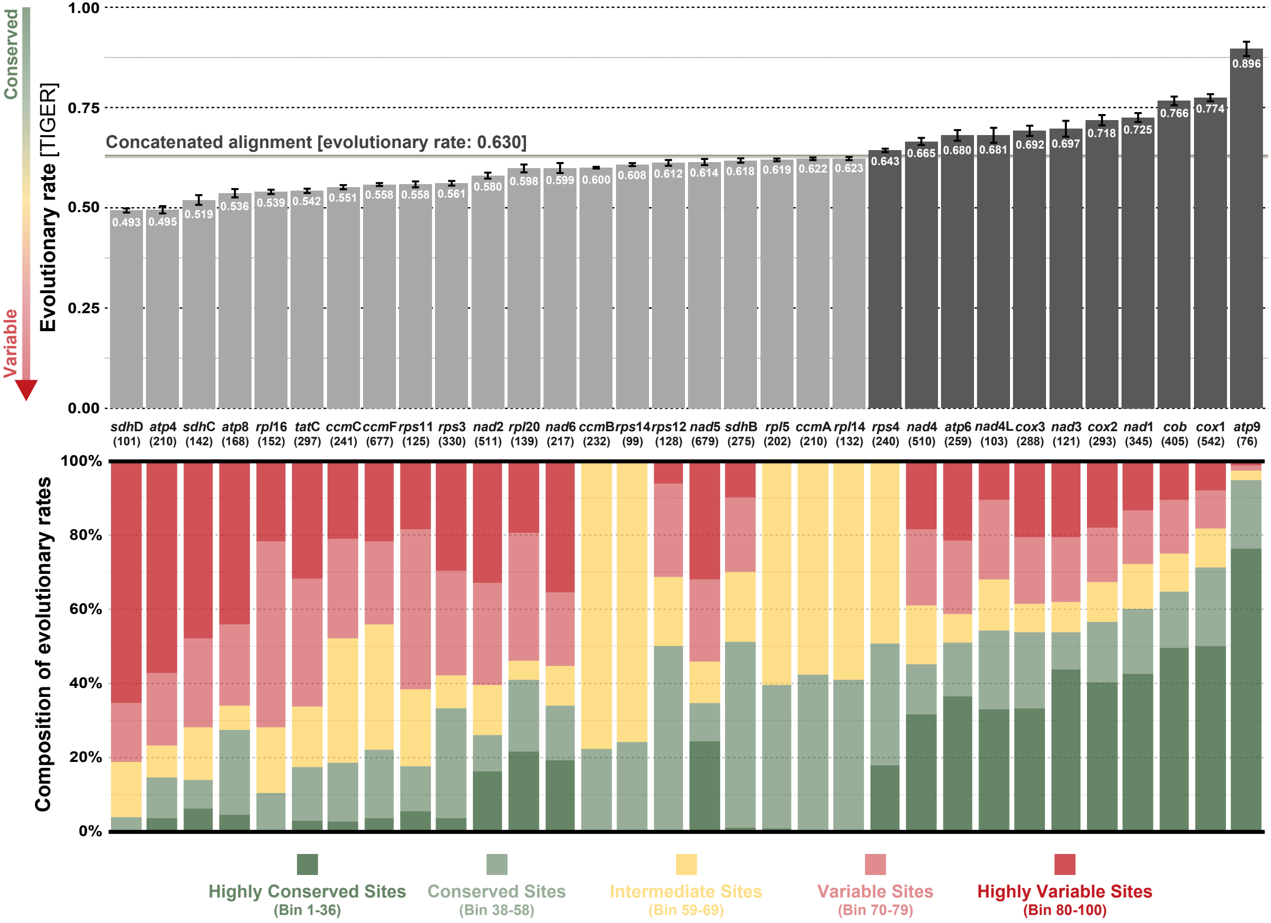

Supplementary Figure S4. Composition of the evolutionary rate of 32 individual genes based on average TIGER values.

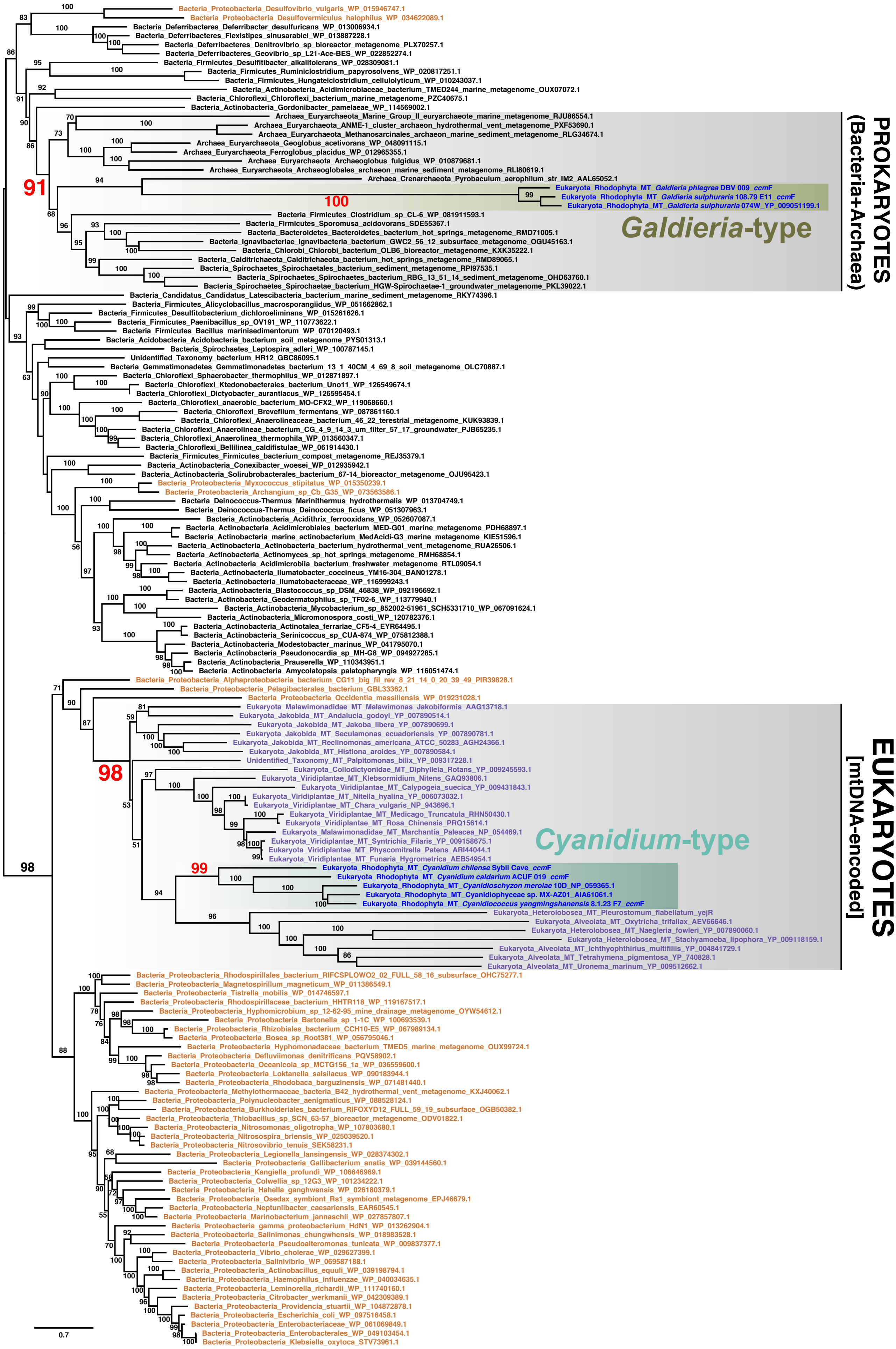

Supplementary Figure S5. The phylogenetic tree of *ccm*F gene using protein sequences.

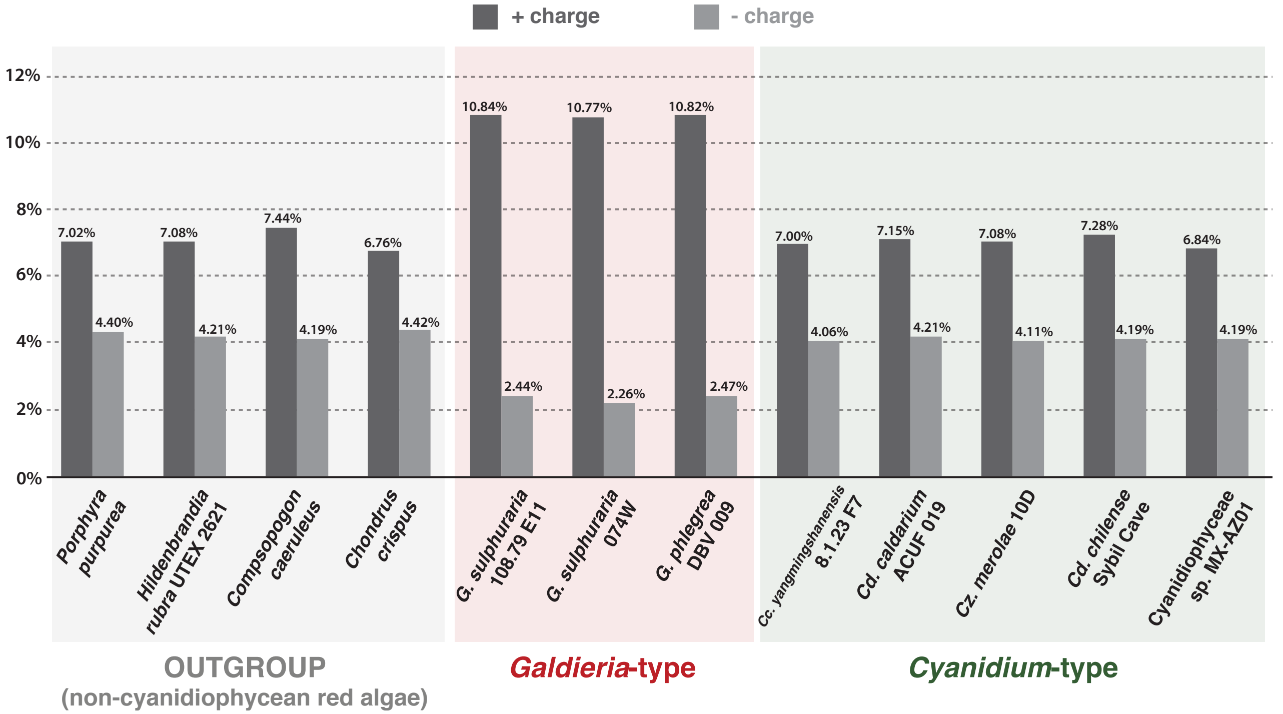

Supplementary Figure S6. Negative and positive amino acids composition of mitochondrial proteins.

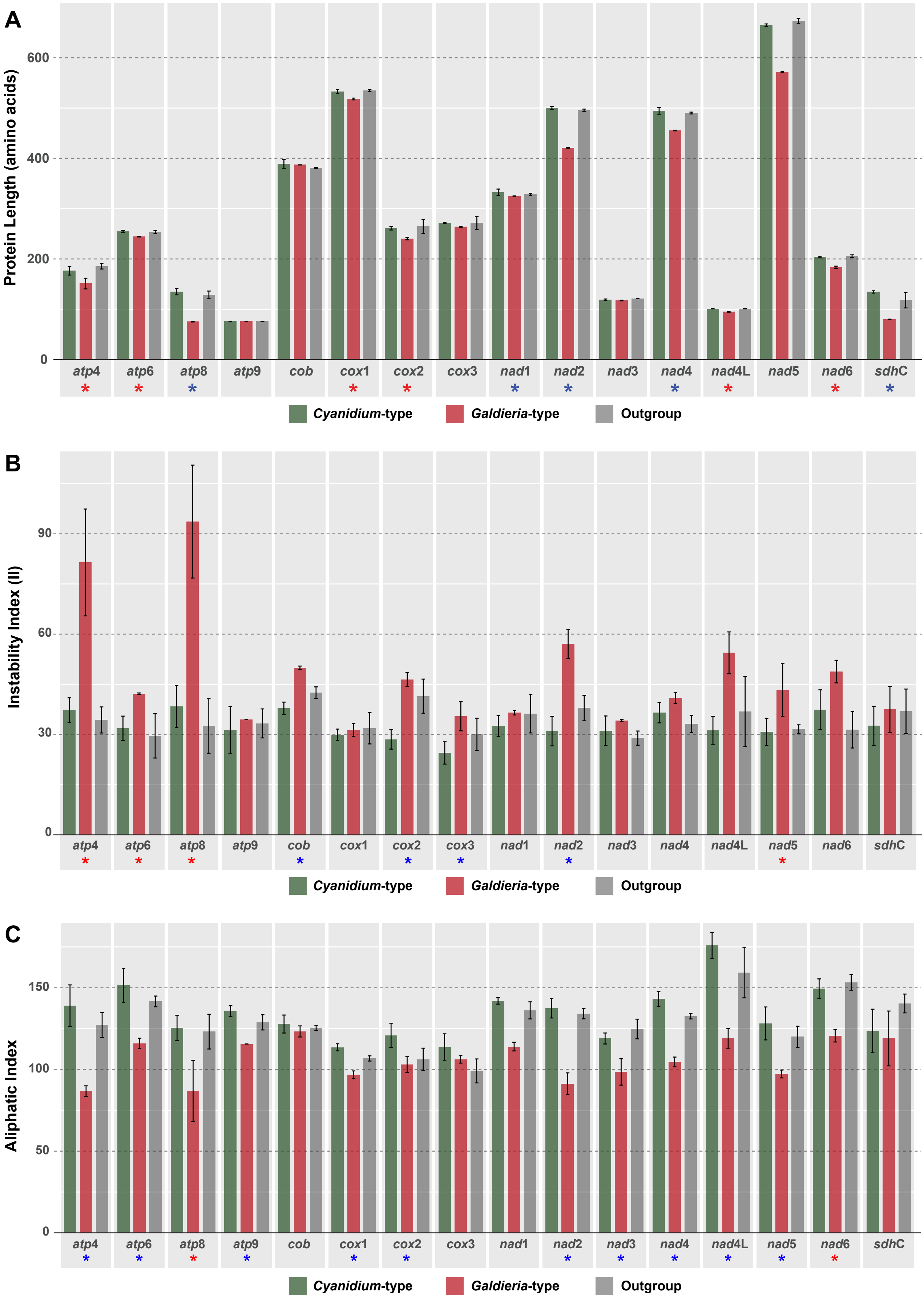

Supplementary Figure S7. Comparison of amino acid features in 16 conserved proteins among three groups (*Cyanidium*-type, *Galdieria*-type, outgroup). (*A*) amino acid length, **(*B*)** instability index (II) is an estimated values of *in vitro* protein stability based on dipeptide composition, **(*C*)** aliphatic index indicates protein thermostability on the basis of aliphatic side chains.

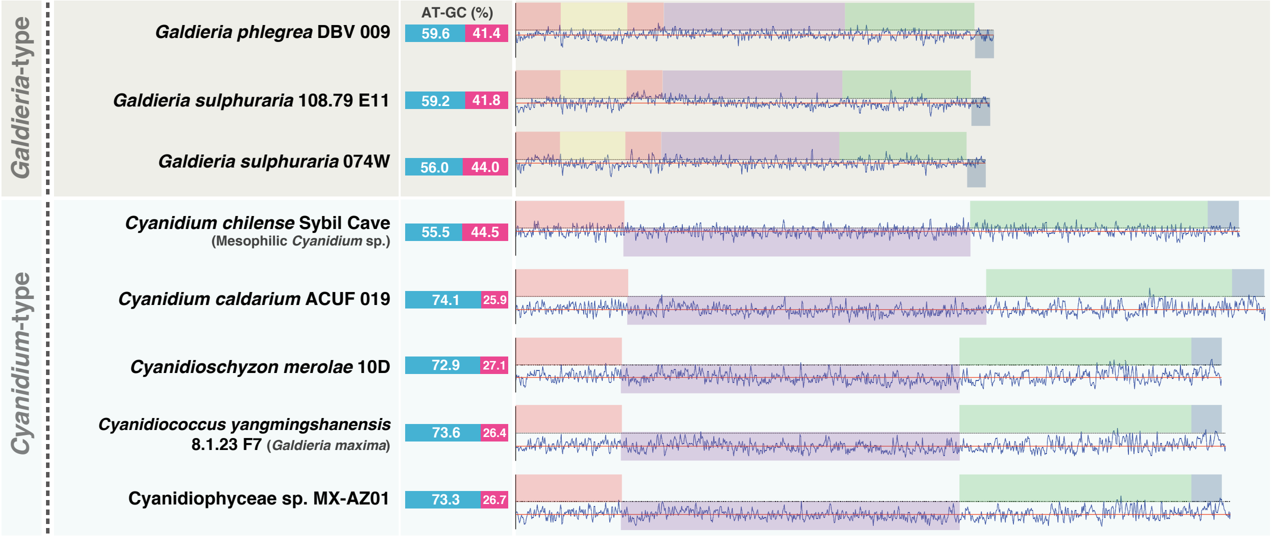

Supplementary Figure S8. GC-contents and synteny comparison of Cyanidiophyceae mitogenomes.

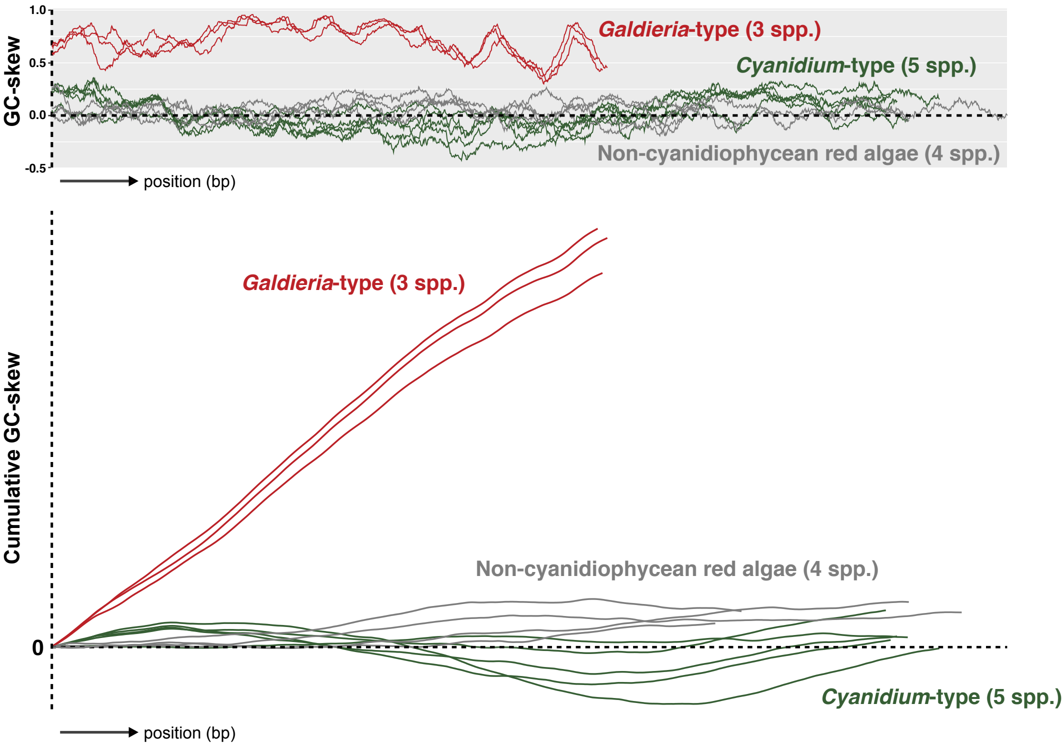

Supplementary Figure S9. GC-skew and cumulative GC-skew of 12 red algal mitogenomes.

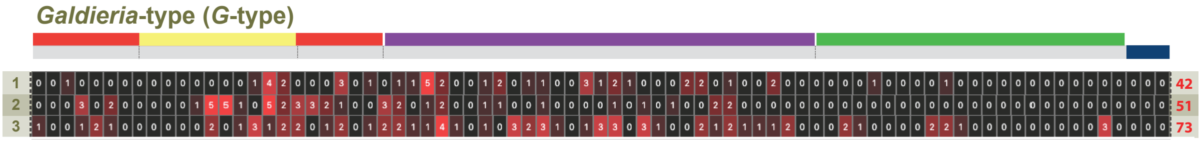

#	G-type/C-type	Strain name	Genome size	# of GC (GC%)	# of quadruplexes	Strands (F/R)	Frequency
1	G-type	*Galdieria phlegrea* DBV 009	21,792	9,014 (41.4%)	42	42/0	1.9/1 kbp
2	G-type	*Galdieria sulphuraria* 108.79 E11	21,611	9,029 (41.8%)	51	51/0	2.4/1 kbp
3	G-type	*Galdieria sulphuraria* 074W	21,428	9,427 (44.0%)	73	73/0	3.4/1 kbp
4	C-type	*Cyanidium chilense* Sybil Cave	33,039	14,698 (44.5%)	9	3/6	0.3/1 kbp
5	C-type	*Cyanidium caldarium* ACUF 019	34,207	8,847 (25.9%)	2	1/1	0.1/1 kbp
6	C-type	*Cyanidioschyzon merolae* 10D	32,211	8,718 (27.1%)	4	2/2	0.1/1 kbp
7	C-type	*Cyanidiococcus yangmingshanensis* 8.1.23 F7	32,387	8,549 (26.4%)	3	1/2	0.1/1 kbp
8	C-type	Cyanidiophyceae sp. MX-AZ01	32,620	8,703 (26.7%)	3	2/1	0.1/1 kbp

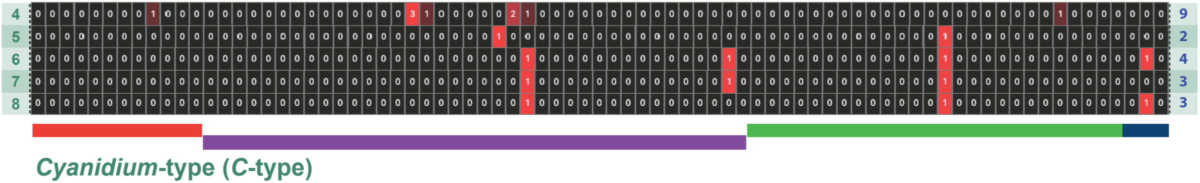

Supplementary Figure S10. Potential G-quadruplexes distributions of eight cyanidiophycean mitogenomes.

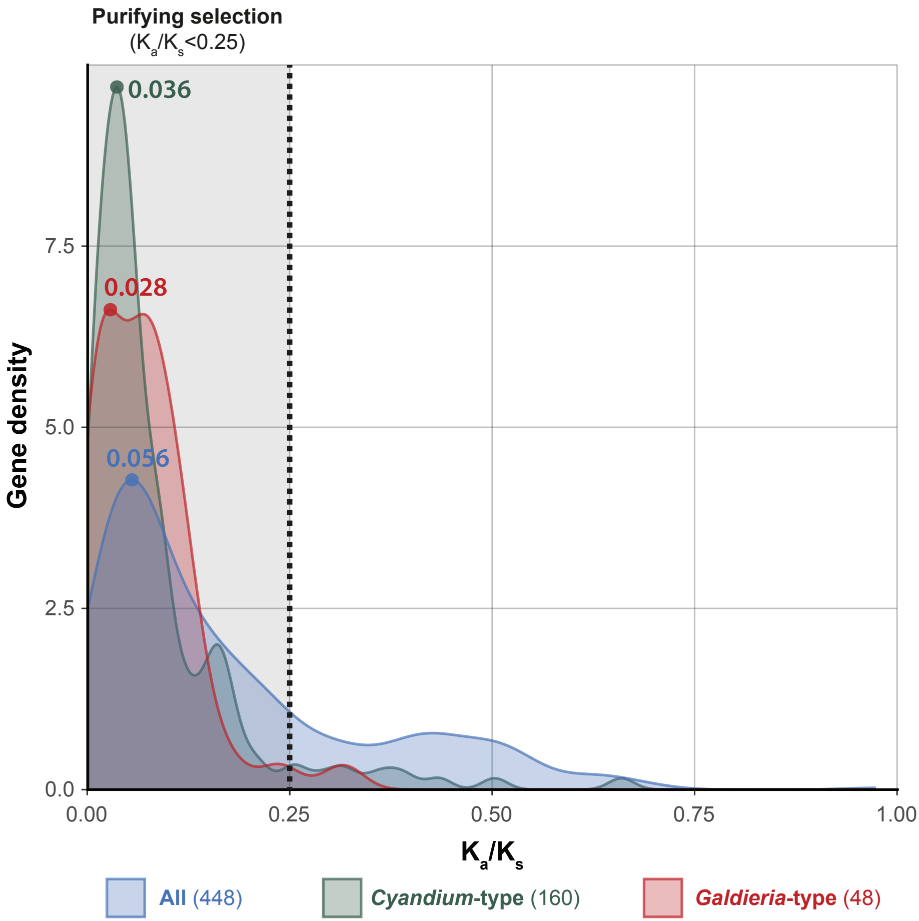

Supplementary Figure S11. Nonsynonymous and synonymous mutations (K_a/K_s) ratio analysis of 16 conserved mitochondrial genes. The numbers in the bracket next to each type are the number of pairs.

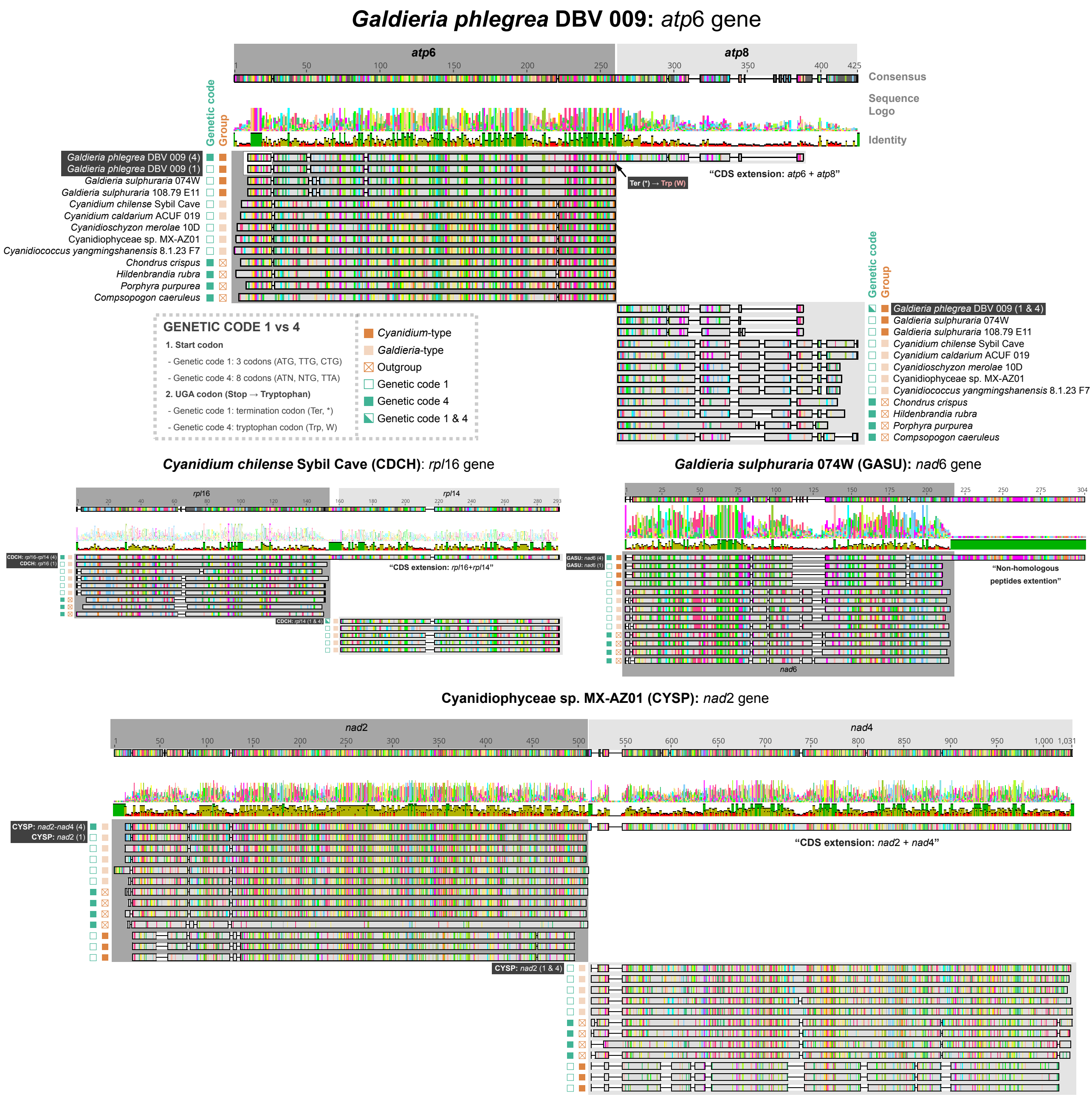

Supplementary Figure S12. Testing of available genetic code for Cyanidiophyceae mitogenomes. Two genetic codes, "Standard Genetic Code 1" and "Protozoan Mitochondrial Genetic Code 4" were used to evaluate the genetic code. A few cases have shown that the use of "Protozoan Mitochondrial Genetic Code 4" extends CDS leading to an invasion of the other CDS.
